# Tree architecture, light interception and water‐use related traits are controlled by different genomic regions in an apple tree core collection

**DOI:** 10.1111/nph.17960

**Published:** 2022-02-08

**Authors:** Aude Coupel‐Ledru, Benoît Pallas, Magalie Delalande, Vincent Segura, Baptiste Guitton, Hélène Muranty, Charles‐Eric Durel, Jean‐Luc Regnard, Evelyne Costes

**Affiliations:** ^1^ AGAP Institut Univ Montpellier, CIRAD, INRAE, Institut Agro 34398 Montpellier France; ^2^ IRHS SFR QuaSaV Université d’Angers, Institut Agro, INRAE 49000 Angers France

**Keywords:** candidate genes, GWAS, high‐throughput field phenotyping, light interception, *Malus domestica*, thermal imaging, T‐LiDAR, tree architecture

## Abstract

- Tree architecture shows large genotypic variability, but how this affects water‐deficit responses is poorly understood. To assess the possibility of reaching ideotypes with adequate combinations of architectural and functional traits in the face of climate change, we combined high‐throughput field phenotyping and genome‐wide association studies (GWAS) on an apple tree (*Malus domestica*) core‐collection.
- We used terrestrial light detection and ranging (T‐LiDAR) scanning and airborne multispectral and thermal imagery to monitor tree architecture, canopy shape, light interception, vegetation indices and transpiration on 241 apple cultivars submitted to progressive field soil drying. GWAS was performed with single nucleotide polymorphism (SNP)‐by‐SNP and multi‐SNP methods.
- Large phenotypic and genetic variability was observed for all traits examined within the collection, especially canopy surface temperature in both well‐watered and water deficit conditions, suggesting control of water loss was largely genotype‐dependent. Robust genomic associations revealed independent genetic control for the architectural and functional traits. Screening associated genomic regions revealed candidate genes involved in relevant pathways for each trait.
- We show that multiple allelic combinations exist for all studied traits within this collection. This opens promising avenues to jointly optimize tree architecture, light interception and water use in breeding strategies. Genotypes carrying favourable alleles depending on environmental scenarios and production objectives could thus be targeted.

## Introduction

Trees have evolved a diversity of architectures enabling their survival, reproduction, and species coexistence under a variety of environmental and ecological conditions (Lines *et al*., 2012; Fournier *et al*., 2013). The architectural variability among and within species results either from changes in organ shape and orientations or tree topological characteristics (Barthélémy & Caraglio, 2007). Genetic polymorphisms associated with tree architectural diversity have been identified through quantitative trait locus (QTL) studies for geometrical (tree height and internode length) and topological (branching rate, proportion of different types of shoots) descriptors, in a number of species including rubber, poplar, spruce and apple (Zhang *et al*., 2006; Kenis & Keulemans, 2007; Segura *et al*., 2007, 2008; Prunier *et al*., 2013; Souza *et al*., 2013). Recent studies have further extended our knowledge of the molecular and hormonal basis of plant architecture (Hollender & Dardick, 2015). The determinism of specific processes such as axillary shoot emergence (Bertheloot *et al*., 2020) or branching angles (Dardick *et al*., 2013) are progressively being deciphered.

Plant architecture is key for resources‐use efficiencies, as it is at the heart of a fundamental trade‐off between carbon gain and water use by impacting leaf area distribution and light interception, water transport and loss, as well as carbon assimilation and allocation. Photosynthesis and transpiration also co‐vary with the aperture and density of stomatal pores (Lawson & Blatt, 2014; see also Liu *et al*., 2012 in apple tree). Over the last two decades, many studies identified contrasting behaviours in the plant’s capacity to maintain their water status upon soil drying (often referred to as isohydric or conservative, i.e. that efficiently maintain high leaf water potential, vs anisohydric or optimistic, i.e. that cannot prevent their leaf water potential from dropping as soil drying develops (Klein, 2014)) whose adaptive value depends on the duration and intensity of the water deficit (Sade *et al*., 2012). In fruit trees, contrasting behaviours in water status maintenance were shown to vary with the season (Franks *et al*., 2007; Lauri *et al*., 2016) and crop load (Naor *et al*., 2008). In grapevine, (an)isohydry was also shown to be genetically controlled in given environmental conditions (Coupel‐Ledru *et al*., 2014). However, the possible interactions between tree architecture and water‐use behaviour in response to soil water deficit have rarely been tackled.

Light interception distribution within the canopy affects the leaf energy balance and consequently leaf transpiration (Monteith, 1965). As such, increasing light interception induces a higher evaporative demand that can increase soil water depletion and severity of the water deficit. Experimental results showing the higher sensibility of larger plants to water deficit are in line with this biophysical regulation (Tisné *et al*., 2010; Rebolledo *et al*., 2013). Stomatal control of transpiration in response to soil water deficit can also interact with canopy microclimate variations (temperature, vapour pressure deficit, radiation) (Willaume *et al*., 2004; Massonnet *et al*., 2008) which induce acclimation of leaves through modifications of carbon or nitrogen related traits (Ngao *et al*., 2020).

Tree architecture can also play a role in leaf water status maintenance through modifications of organ topological connections and xylem water flow (Schultz, 2003; Lauri *et al*., 2011). Nevertheless, it is unknown to which extent these relationships between plant functioning and architecture could be modified in large populations of individuals with contrasted genetic backgrounds. Fruit trees are relevant species to address this question because they naturally display large architectural genotypic variability and within‐canopy microclimate variation (Costes & Gion, 2015).

Overall, the possibility of reaching ideotypes with optimal combinations of architectural and functional traits under limiting soil water conditions remains challenging given the biophysical interactions between both types of traits, and the lack of knowledge about their possible common genetic control. In fruit trees, the recent development of core‐collections representative of the existing cultivated diversity (e.g. Belaj *et al*., 2012; Lassois *et al*., 2016; Garcia‐Lor *et al*., 2017) and the availability of very high‐throughput genotyping (e.g. Bianco *et al*., 2016) open new avenues to tackle these questions across a wide genetic diversity. The recent development of high‐throughput phenotyping methods and of experimental orchards appropriately designed (Jung *et al*., 2020) offer new opportunities for evaluating large populations of individuals at multi‐scales and for many targeted traits (Araus & Cairns, 2014).

In a study performed on a bi‐parental population of apple trees under contrasted watering scenarios, Virlet *et al*. (2015) identified QTLs associated either with vegetative development or canopy surface temperature using indicators derived from airborne imaging. More recently, Santini *et al*. (2021) identified putative genes associated with vegetation indices and leaf temperature from unmanned aerial vehicle (UAV) flights using genome‐wide association studies (GWAS) on a *Pinus halepensis* collection. Nevertheless, these studies did not integrate any characterization of plant architecture to analyse phenotypic or genotypic correlation between tree architecture and response to water deficit.

In a recent work (Coupel‐Ledru *et al*., 2019), we deployed a combination of high‐throughput phenotyping methods on an apple tree core‐collection (241 cultivars) mostly consisting of French local or old dessert apple cultivars. In this study, architectural traits were computed making use of the light detection and ranging (LiDAR) sensing technology which exploits laser light to detect and measure distances of objects. Terrestrial LiDAR (T‐LiDAR) scans of individual trees were thus analysed to compute architectural traits such as tree leaf area, silhouette to leaf area ratio (STAR) as a proxy of light interception or hulls describing three‐dimensional (3D) canopy volumes (Coupel‐Ledru *et al*., 2019; Pallas *et al*., 2020). Together with T‐LiDAR scans, airborne multispectral and thermal images were acquired and compared to *in planta* measurements (Coupel‐Ledru *et al*., 2019) to achieve phenotyping of both architectural and functional traits. Here, we extend this high‐throughput multi‐scale characterization of the core‐collection by deploying it under increasing soil drying and coupling it with GWAS. Multivariate analysis and GWAS performed with single nucleotide polymorphism (SNP)‐by‐SNP and multi‐SNP methods showed that vegetative development, light interception and canopy surface temperature response to soil drying are ruled by independent genetic controls. These results reveal that maximizing light interception at the plant scale through architectural characteristics could be achieved without impairing their response to water resource availability.

## Materials and Methods

### Setup and plant material

The study was performed in 2017 on an apple tree (*Malus domestica*) core‐collection of 241 cultivars (Lassois *et al*., 2016; list in Coupel‐Ledru *et al*., 2022), all grafted onto M9 Pajam®2 rootstock, and grown in field conditions at INRAE experimental unit ‘DiaScope’ (43°36′N, 03°58′E, near Montpellier, France). Four trees per cultivar were planted in 2014 (4 yr‐old at the beginning of the experiment), yielding a total of 964 trees. All trees were left unpruned. During the first 2 yr after planting, all trees were grown under nonlimiting irrigation (provided by micro‐sprayers located between‐trees). Differentiated water regimes were set up during summers 2016 and 2017, with two well‐watered (WW) and two water‐deficit (WD) trees per cultivar. The orchard comprised 10 rows of 100 trees, embedding two replications of two trees per genotype randomly distributed within the field. WW and WD tree rows alternated within the trial, one WW tree facing one WD tree for each genotype. Measurements were performed in summer 2017. On 7 July, irrigation was withheld on WD rows until the end of the month. Dynamics of soil drying were evaluated measuring soil water potential (*ψ*_soil_) with Watermark® tensiometric probes (−30 and −60 cm depth) randomly distributed within the orchard at the bottom of 13 trees. Throughout the experiment, *ψ*_soil_ remained constant for WW trees (*c*. −8 kPa) while it gradually decreased for WD trees and reached on average −72 kPa on 27 July (Supporting Information Fig. S1). All trees were manually thinned to one fruit per inflorescence and fertilizers were applied to avoid any mineral deficiency.

### Airborne imaging and computation of thermal and vegetation indices

Airborne images were acquired on 5, 12, 17 and 27 July (see Table S1 for the atmospheric conditions during flights). Flights were carried out using a Mikrokopter® hexa‐rotor drone. Thermal and multispectral images were acquired, respectively, by a FLIR®TAU2.7 uncooled camera (7.5–13 μm, 640 × 512 pixels resolution) and an AirPhen.v.3 camera measuring reflectance in blue, green, red, red‐edge, and near‐infrared (NIR) with a bandwidth close to 10 nm (Coupel‐Ledru *et al*., 2019). At each date, three consecutive flights at 25 m height were performed to cover the entire field (1.2 ha). Based on global positioning system (GPS) coordinates, a 0.70 m radius zone was delineated around the centre of each tree. All thermal and multispectral variables – NDVI (Rouse, 1974), GNDVI (Gitelson *et al*., 1996) and MCARI2 (Haboudane *et al*., 2004) – were computed on this zone for each tree. Mean canopy surface temperature (TS) was computed, and differences between canopy surface and air temperature (TA) were calculated (TSTA) to account for potential thermal variations between dates.

At the flight dates, soil dryness increased on WD rows (Fig. S1): 5 July, no WD (*ψ*_soil,mean_ = −8 kPa); 12 July, light WD (*ψ*_soil,mean_ = −23 kPa); 17 July, moderate WD (*ψ*_soil,mean_ = −48 kPa); 27 July, severe WD (*ψ*_soil,mean_ = −72 kPa). Predawn leaf water potential (*ψ*_leaf,predawn_) and midday leaf (*ψ*_leaf,midday_) and stem (*ψ*_stem,midday_) water potentials were also measured on 45 trees across the orchard, with two Schölander pressure chambers previously cross‐calibrated using a distributed, pressurized nitrogen source (Table S2; predawn and midday measurements respectively took place between 02:30 and 03:15 UTC, and between 11:00 and 14:00 UTC). For WW trees, *ψ*_stem,midday_ and *ψ*_leaf,midday_ were lower on 12, 17 and 27 as compared to 5 July, likely due to drier atmospheric conditions. Consistently with *ψ*_soil_, *ψ*_stem_ and *ψ*_leaf_ showed decreasing values for WD trees from the onset of irrigation withholding to the end of the experiment (from −0.54 MPa to −1.85 MPa for *ψ*_stem,midday_).

### T‐LiDAR and derived architectural variables

Two measurements series were performed with a T‐LiDAR scanner. The first took place in October 2017 on leafy trees, to collect ‘summer’ variables (as described in Coupel‐Ledru *et al*., 2019), and the second in February 2018 for ‘winter’ variables (not previously presented) (Fig. S2a). Scans were carried out with a 360° view each ten meters on every row with an angular resolution of 0.04°. During each measurement series, 200 scans were used to phenotype the whole collection. Each individual tree point cloud was analyzed with PlantScan3D (Boudon *et al*., 2014) and PlantGL (Pradal *et al*., 2009) in OpenAlea (Pradal *et al*., 2008). Summer variables provided information on the canopy structure: alpha and convex hull volumes (a_volume, c_volume) and plant height (height). c_volume represents the maximal space occupation of the tree, whereas a_volume represents the volume filled by leaves within this convex hull. For assessing light interception variables, we computed the STAR (Sinoquet *et al*., 2001). A convexity index (ci) correlated with STAR (Coupel‐Ledru *et al*., 2019) was computed as the ratio a_volume/c_volume. Winter scans were used to estimate the number of axes (nb_axes) and the total cumulative axis length (total_length) per tree as in Pallas *et al*. (2020).

### Manual measurements

In autumn 2017, trunk circumference was manually measured on each tree and used to compute trunk cross‐sectional area (TCSA) assuming a cylinder trunk shape. TCSA is reported to be a good indicator of tree size (Strong & Azarenko, 2000).

### Computation of genotypic values and genotypic responses to soil water deficit

All data analyses were performed using R (R Development Core Team, 2020). In order to calculate synthetic variables, a principal component analysis (PCA) was performed (R/factominer) on the variables related to tree architecture using individual tree values. Tree coordinates on the two first PCA dimensions (respectively representative of vegetative development and light interception capacity) were further used as additional traits.

Different mixed‐effect models were tested (R/lme4) with a random effect of the genotype (*G*), fixed effects of the watering scenario (*S*), spatial design (row), date of measurements (*D*), and flight within each date (*F*). For T‐LiDAR variables, a fixed‐effect of the tree position relative to the LiDAR (*R*) was included. The best model was selected (Table 1) to minimize the Akaike Information Criterion (AIC) (Table 1). Variance components were used to estimate broad‐sense heritability (*H* ^2^) as:

$$H^{2}=\text{var}G/\left(\text{var}G+\text{varRes}/n\right)$$

with var*G* the genotypic variance, varRes the residual variance, and *n* the number of replicates per genotype.

**Table 1:** Statistical indicators of the genotypic variability for vegetation indices and vegetative architecture‐related traits on a collection of 241 apple tree cultivars.

Measurement type	Trait	Broad sense *H* ^2^	*H* ^2^ CI	Selected model	Variance		Minimum	Mean	Maximum	CV	Genomic *h* ^2^
					Genotypic (var*G*)	Residual (varRes)					
Multi‐spectral imagery	NDVI	0.94	0.93–0.94	*P = G + S + D + F*	0.0080	0.0053	0.17	0.42	0.6	0.17	0.41
	GNDVI	0.94	0.93–0.94	*P = G + S + D + F*	0.0040	0.0027	0.11	0.3	0.44	0.17	0.44
	MCARI2	0.92	0.92–0.93	*P = G + S + D + F*	0.025	0.014	0.18	0.55	0.82	0.22	0.35
T‐LiDAR data	c_volume	0.82	0.80–0.84	*P = G*	1.02	0.93	0.79	3	5.72	0.3	0.52
	a_volume	0.77	0.75–0.79	*P = G + S*	0.053	0.064	0.24	0.79	1.55	0.25	0.49
	ci	0.86	0.85–0.88	*P = G + S*	0.061	0.038	0.14	0.29	0.51	0.25	0.56
	STAR	0.79	0.78–0.81	*P = G + S*	0.011	0.011	0.55	0.73	1.06	0.12	0.27
	height	0.81	0.79–0.83	*P = G + S*	0.070	0.066	1.45	2.25	2.91	0.11	0.64
	total_length	0.80	0.78–0.81	*P = G + R*	96.1	98.1	9.49	30.64	60.39	0.28	0.57
	nb_axes	0.83	0.82–0.85	*P = G + R*	8528	6890	70.12	219.46	545.38	0.38	0.65
Manual measurement	TCSA	0.71	0.69–0.73	*P = G + S*	13.1	21.4	9.5	16.0	18.9	0.15	0.37
PCA coordinates	Dim.1	0.77	0.77–0.81	*P = G + R*	1.37	1.61	−3.62	0	3.23	–	0.54
	Dim.2	0.85	0.83–0.85	*P = G + S*	1.15	0.78	−2.61	0	2.99	–	0.38

Genotypic (var*G*) and residual (varRes) variance components were estimated with the mixed‐effect model accounting for random genotype effect (*G*) and for fixed effects among watering scenario (*S*), date of measurements (*D*), position of the tree for T‐LiDAR measurements (*R*), and drone flight within each day of measurements for airborne imaging (*F*). They were used to estimate a broad‐sense heritability such as *H* ^2^ = var*G*/(var*G* + varRes/*n*). *H* ^2^ 95% confidence interval (CI) was determined by bootstrap. Mean, minimum, maximum and coefficient of variation (CV) values were calculated using best linear unbiased predictors (BLUPs) (*n* = 241 cultivars). Genomic heritability (*h* ^2^) was retrieved from MLMM outputs. a_volume, alpha hull volume (m^3^); c_volume, convex hull volume (m^3^); ci, convexity index; STAR, silhouette to leaf area ratio; height, plant height (m); total_length, total cumulative axis length (m); nb_axes, number of axes; TCSA, trunk cross‐sectional area (cm²); Dim.1 and Dim.2, respectively the coordinates of first and second principal components of the principal component analysis (PCA) on light interception and vegetative architecture traits.

A 95% confidence interval (CI) for *H*² was calculated using parametric bootstrap (1000 simulations) with the function BootMer (R/lme4).

Best linear unbiased predictors (BLUPs) of genotypic values were extracted for each trait. To quantify the genotypic response to soil water deficit, we calculated at each date another variable named resp.TSTA computed as the difference between BLUPs of TSTA under WD condition (TSTA.WD) and BLUPs of TSTA under WW condition (TSTA.WW). The ratios resp.TSTA_12.07/resp.TSTA_27.07 (named contrib_12.07) and resp.TSTA_17.07/resp.TSTA_27.07 (contrib_17.07) were further calculated to quantify the proportion of variation in resp.TSTA observed at severe soil dryness intensity (27 July) that was achieved at light (12 July) and moderate (17 July) intensities of soil dryness.

Multivariate analyses of correlations between all traits were performed on BLUPs.

### Genome‐wide association studies (GWAS)

#### Association tests

GWAS was performed to look for associations between BLUPs and genomic markers. We used the Axiom®Apple 480 K array that resulted in 275 223 robust SNPs for GWAS upon stringent filters (Bianco *et al*., 2016; Denancé *et al*., 2022). Filtering for minor allelic frequency (MAF) above 0.05 yielded 262 437 SNPs actually tested in GWAS. All results use the SNP positions on the latest version (v.1.1) released for the apple genome based on the doubled haploid GDDH13 (Daccord *et al*., 2017).

Two types of GWAS models were used. A SNP‐by‐SNP model implemented in gemma v.0.97 (Zhou & Stephens, 2012) based on a linear mixed model was used to test the significant effect of each SNP, as follows:

Y=μ+Xβ+U+E

where *Y* is the vector of BLUPs, *μ* the overall mean, *X* is the vector of SNP scores, *β* is the additive effect, *U* and *E* represent random polygenic and residual effects, respectively. The variance‐covariance matrix of *U* was determined by a genetic relatedness (kinship) matrix estimated based on SNPs with MAF > 0.05, using VanRaden kinship estimator (VanRaden, 2008). Genomic heritabilities (*h*²) were computed as the ratio of variance explained by the polygenic effects (*G*) to the total BLUP variance, in the null GWAS model, i.e. without considering the effect of individual SNPs (*β*). This model, accounting only for relatedness, was found (based on AIC values) to be the best to control confounding factors as compared to models accounting for structure (assessed with the Admixture software (Alexander & Lange, 2011) and found to be weak), or for relatedness plus structure. The SNP effects were considered significant when the *P*‐value (*P*val) from the Wald test statistic was smaller than the Bonferroni threshold, controlling the family‐wise error rate at 5% to account for multiple testing. This threshold was calculated on a subset of nearly independent SNPs using a LD pruning method implemented in Plink/indep (Purcell *et al*., 2007) with a recommended set of parameters (window of 50 SNPs sliding by five SNPs and a variance inflation factor of two). This yielded 23 200 independent SNPs hence a genome‐wide significance threshold of −log_10_(*P*val) = 5.63.

We also used the multi‐locus mixed model (MLMM) proposed by Segura *et al*. (2012). The model is based on a stepwise regression procedure and jointly analyses all SNPs in order to select a subset of SNPs with large effects while handling LD. This procedure starts with a SNP‐by‐SNP model, followed by inclusion, at every iteration, of the SNP with the smallest *P*‐value as an additional fixed effect, until the proportion of variance explained by the polygenic effect is close to zero. We fitted it with R/mlmm allowing a maximum of seven iterations. The selected model was the one with the largest number of SNPs, which all have a *P*‐value below the multiple‐testing significance threshold as previously determined (mBonf criterion, Segura *et al*., 2012).

For each trait, GWAS results obtained with gemma and MLMM were compared. SNPs selected by both methods were considered ‘highly reliable’ and further used for analysing interaction effects and candidate genes. R/rutilstimflutre (Flutre, 2019) was used for post‐processing GWAS results (e.g. boxplots of allelic effects).

#### Effects of SNPs identified as cofactors and analyses of interactions between significant SNPs

For each trait, the total percentage of variation explained by all significant SNPs identified with MLMM was extracted from MLMM output. The percentage of explained variance and the effect associated to each individual SNP was also assessed, with linear models for each SNP separately. The ‘mode’ of action of each SNP was computed as the ratio of dominance to additive effect as described in Urrestarazu *et al*. (2016). No dominance effect is associated with ratio values close to 0 whereas values close or > 1 are associated with strong dominance.

When several ‘highly reliable’ SNPs were detected for a given trait, all possible pairwise interactions were tested. More specifically, we performed generalized‐least squares *F*‐tests between the models including additive and interaction effects between SNPs or only additive effects (like in MLMM). For the particular situation of chr13 where significant SNPs were detected for several traits, a haplotype‐based analysis was attempted to decipher the genetic control of these traits (Methods S1).

#### Intervals definition and candidate genes exploration

In order to investigate candidate genes in the associated regions, QTLs were defined as intervals of ±100 kb around the most significant SNPs (in line with Urrestarazu *et al*., 2016). We retrieved the list of genes within the intervals together with their annotations of protein‐coding and non‐protein‐coding genes using GDDH13 genome v.1.1 browser (https://iris.angers.inra.fr/gddh13/). Annotations for gene biochemical functions were enriched by the biological functions inferred from the putative orthologues identified in *Arabidopsis thaliana* retrieved from (www.rosaceae.org). We searched UniProt (www.uniprot.org/) and TAIR (www.arabidopsis.org/) databases to get a complete description of the gene functions based on gene ontology and annotation.

## Results

### Strong genotypic variability for multi‐spectral indices and vegetative architecture

At each date, NDVI, GNDVI and MCARI2 showed large variability with bell‐shaped almost symmetric distributions (Fig. S3) and a low effect of WD (e.g. WD decreased average NDVI as compared to WW conditions by < 1% on 17 July and by < 5% on 27 July). Importantly, strong positive correlations (*r* > 0.7) were observed between dates. We thus estimated a single BLUP for subsequent analyses for each of these three indices, integrating the four dates and both scenarios. Corresponding *H* ^2^ values were higher than 0.9 (Table 1). All three indices displayed a wide range of variation between cultivars, e.g. up to a six‐fold difference between minimum and maximum BLUPs for NDVI (Table 1).

**Fig. 1:**
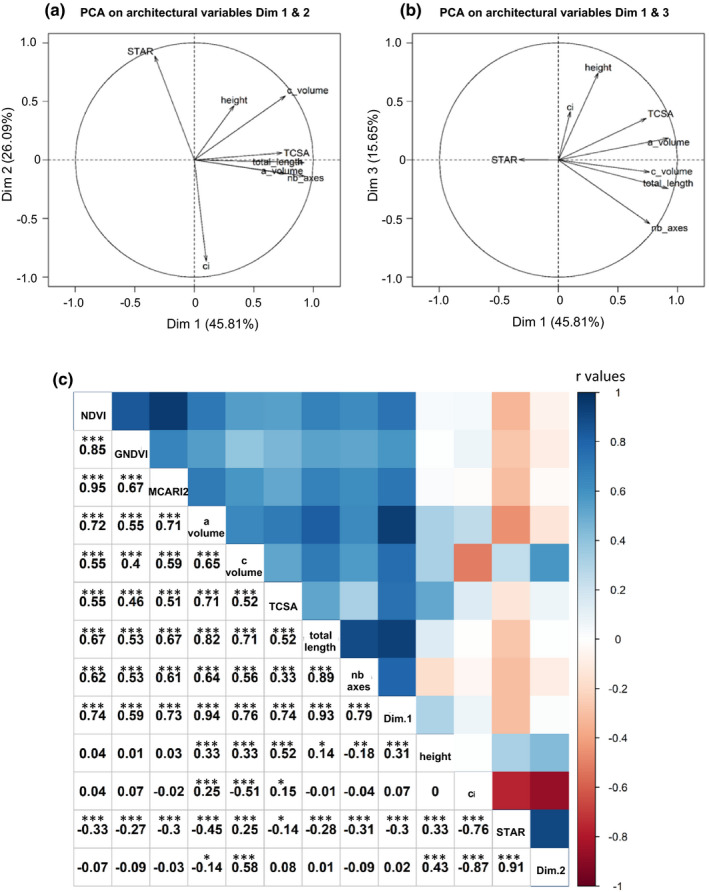
Principal component analysis (PCA) on light interception, plant architecture and shoot traits, and matrix of correlations between genotypic values of the vegetation indices and vegetative architecture‐related traits in an apple tree collection. (a, b) Projection of variables on the first and second (respectively first and third) dimensions (‘Dim’) of the PCA. Percentages of variance explained by each axis are displayed in the axis label. The analysis was carried out on individual tree values. *n* = 964 trees. (c) Matrix of correlations calculated from best linear unbiased predictors (BLUPs) of genotypic values for *n* = 241 cultivars. In the bottom left part of the matrix, Pearson’s correlation coefficients (*r* values) are indicated together with their significance (***Pval < 10^−3^,**Pval < 10^−2^, *Pval < 0.05, no star not significant). In the top right part of the matrix, the colours of the squares are used to represent these *r* values. a_volume, alpha hull volume; c_volume, convex hull volume; ci, convexity index; STAR, silhouette to leaf area ratio; height, plant height; total_length, total cumulative axis length; nb_axes, number of axes; TCSA, trunk cross‐sectional area; Dim.1 and Dim.2, respectively first and second principal components of the PCA analysis on light interception, plant architecture and shoot traits.

Canopy development, tree architecture and light interception related traits were computed at a single date, in either summer or winter. The models selected sometimes accounted for the watering scenario but its effect remained low (Table 1; e.g. a_volume was decreased by a 5% in WD trees as compared to WW trees). This effect likely resulted from cumulative effects of summer water depletion in 2016 and 2017. A wide range of genotypic variability was found for all traits (Table 1), revealing a strong diversity within the collection for both canopy and tree structure (e.g. a_volume ranged from 0.24 to 1.55 m^3^ and nb_axes between 70 and 545) and light interception (e.g. STAR varied from 0.55 to 1.06). High values of *H*² were observed (0.71–0.86, Table 1). The first two dimensions of the PCA jointly explained 72% of the total variability (Fig. 1a,b). The first dimension (Dim.1) was associated with vegetative development (a_volume, TCSA, nb_axes, total_length) whereas the second (Dim.2) was associated with light interception (ci, STAR) (Fig. 1a,b and Fig. S2b,c). The *H* ^2^ values for Dim.1 and Dim.2 were high (0.77 and 0.85).

Genotypic correlations (Fig. 1c) emphasized the strong correlations between variables related to vegetative architecture on the one hand, or light interception on the other hand. No clear correlations between both groups were observed except for STAR that was slightly negatively correlated to architectural traits (a_volume, c_volume). NDVI was strongly correlated with GNDVI and MCARI2 (*r* = 0.85 and 0.95, respectively) while the correlation between GNDVI and MCARI2 was lower (*r* = 0.67). BLUPs of these three variables were significantly, positively correlated with the ones related to canopy development, particularly a_volume and Dim.1 (Fig. 1c).

### Genotype‐dependent control of canopy temperature in WW conditions

TSTA, the difference between canopy surface and air temperature computed from thermal imaging, was first evaluated for the WW trees as a proxy for water loss in nonlimiting watering conditions (TSTA.WW). Despite the stability of *Ψ*_soil_ across the four measurement dates (Fig. S1), mean values of TSTA.WW differed between dates (Fig. S4), likely due to atmospheric variations between dates (Table S1). However, the genotype had a significant effect at each date (*P*val < 10^–3^). To estimate BLUPs of TSTA.WW independent of atmospheric variations, we used data collected on the 4 d with a mixed‐effect model including a fixed effect of the measurement date. These TSTA.WW BLUPs displayed a substantial range of variability among the cultivars (0.4–2.3°C) with *H* ^2^ = 0.67 (CI 0.65–0.68). This suggests a genotype‐dependent control of water loss in WW conditions.

### Large genotypic variations in the response of canopy surface temperature to intensifying soil drying

**Fig. 2:**
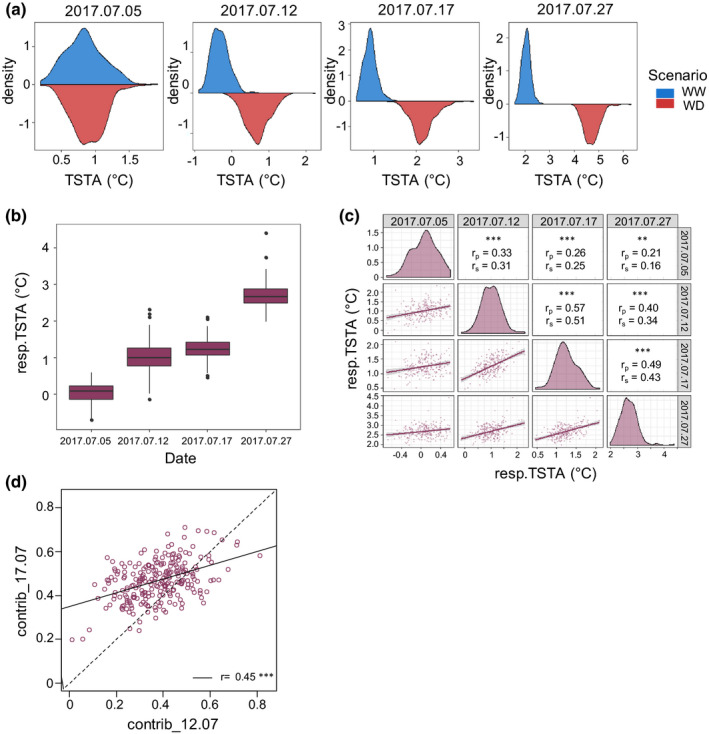
Canopy surface temperature (TSTA) variability and increase with soil drying within an apple tree collection of 241 cultivars. (a) Density plots of TSTA in the well‐watered (WW) and water‐deficit (WD) scenarios in July. (b) Boxplots representation of resp.TSTA (difference between best linear unbiased predictors (BLUPs) of TSTA.WD and of TSTA.WW), on the four measurement dates. Boxplots present the median (centre horizontal line) and interquartile range (purple box) of each group. The upper and lower whiskers represent data within 1.5 × the interquartile range, and values beyond these upper and lower bounds are considered outliers, marked with dots. (c) Graphs of correlations between resp.TSTA across dates with their associated Pearson’s (*r*_p_) and Spearman’s (*r*_s_) correlation coefficients (***, *P* < 10^−4^; **, *P* < 10^–2^). (d) Correlation between contrib_12.07 and contrib_17.07, respectively the ratios resp.TSTA_12.07/resp.TSTA_27.07 and resp.TSTA_17.07/resp.TSTA_27.07. *n* = 241 varieties.

Genotypic values of TSTA for WD trees (TSTA.WD, Fig. 2) were calculated at each date. The *H*² values were similar between dates (5 July, *H* ^2^ = 0.30 with CI = 0.25–0.33; 12 July, *H* ^2^ = 0.43 with CI = 0.39–0.48; 17 July, *H* ^2^ = 0.44 with CI = 0.40–0.49; 27 July: *H* ^2^ = 0.35 with CI = 0.31–0.39). TSTA.WD displayed a marked and progressive increase along with intensification of soil drying (Fig. 2a). Mean resp.TSTA increased over the experiment (Fig. 2b): from 1°C under light WD up to 2.7°C under severe WD. A wide range of variation was found among the 241 cultivars for resp.TSTA (Fig. 2b,c) which ranged from 0 to 2.3°C at the onset of soil WD and from 1.9 to 4.4°C under severe WD. The resp.TSTA genotypic values were significantly correlated between consecutive dates, but correlations were lower when dates were more distant in time (Fig. 2c). Besides, contrib_12.07 and contrib_17.07 were positively correlated with higher values for contrib_17.07 for a large majority of varieties (Fig. 2d). This indicates a general consistency in the ranking of genotypes for the proportion of their maximal canopy temperature increase observed at the light and moderate intensities of soil dryness. Yet, the correlation was not tight (*r* = 0.45), and contrib_12.07 and contrib_17.07 did not correlate with the absolute value of maximal resp.TSTA observed on 27 July (Fig. S5). These results emphasize that genotypes prematurely affected by WD are not necessarily those responding the stronger to soil dryness.

### Genotypic variations in canopy surface temperature and vegetative architecture are weakly related

**Fig. 3:**
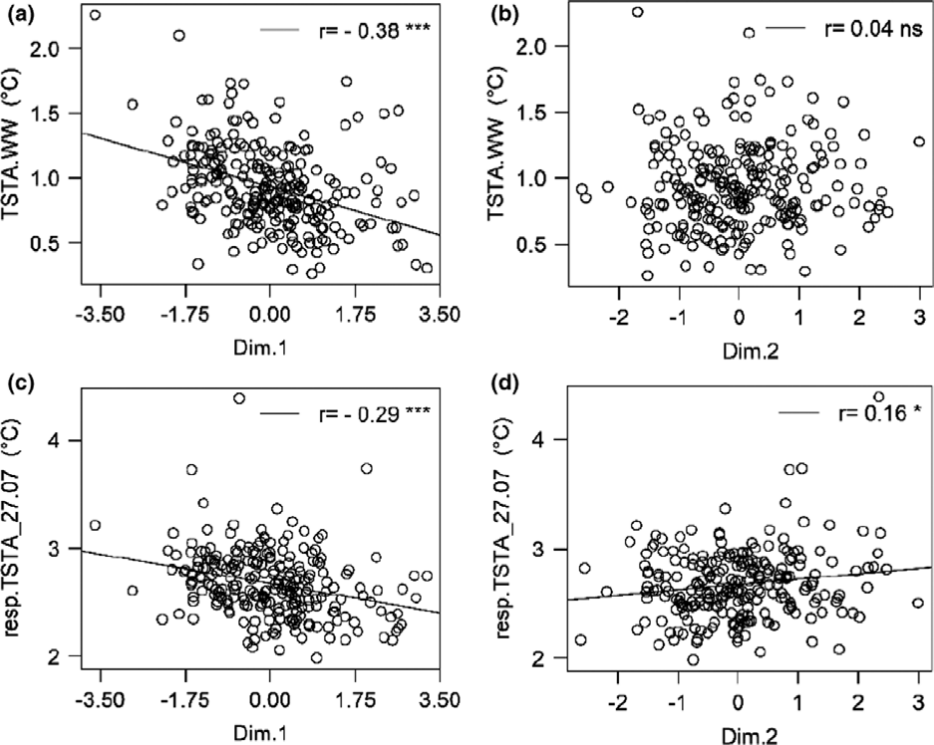
Correlations between canopy surface temperature and traits related to vegetative architecture and light interception in an apple tree collection of 241 cultivars. (a, b) Relationship between best linear unbiased predictors (BLUPs) of canopy temperature in well‐watered conditions (TSTA.WW), and vegetative development as represented by Dim.1 (a), or light interception efficiency as represented by Dim.2 (b). (c, d) Relationship between BLUPs of the response of canopy temperature to severe water deficit (resp.TSTA_27.07), and vegetative development (Dim.1) or light interception efficiency (Dim.2). *n* = 241 cultivars. Pearson’s correlation coefficients are displayed together with their significance (***, *P* < 10^−3^; *, *P* < 0.05; ‘ns’, nonsignificant).

We further investigated correlations between BLUPs for traits related to canopy development or light interception on the one hand, and canopy surface temperature under WW (TSTA.WW) or in response to severe WD conditions (resp.TSTA_27.07) on the other hand (Figs 3, S6). Correlations were generally low. A negative trend was observed between *TSTA.WW* (respectively resp.TSTA_27.07) and canopy development, suggesting a tendency for more vigorously growing cultivars to maintain stronger canopy temperature regulation than small cultivars. However, the weakness of these correlations (*r* < 0.43) indicated that multiple combinations of these traits exist depending on the cultivar. Regarding the relationship between canopy surface temperature (either TSTA.WW or resp.TSTA_27.07) and light interception efficiency, correlations appeared even weaker (0.04 < *r* < 0.26), although significantly different from zero.

### Different genomic regions are associated with tree architecture and light interception

**Fig. 4:**
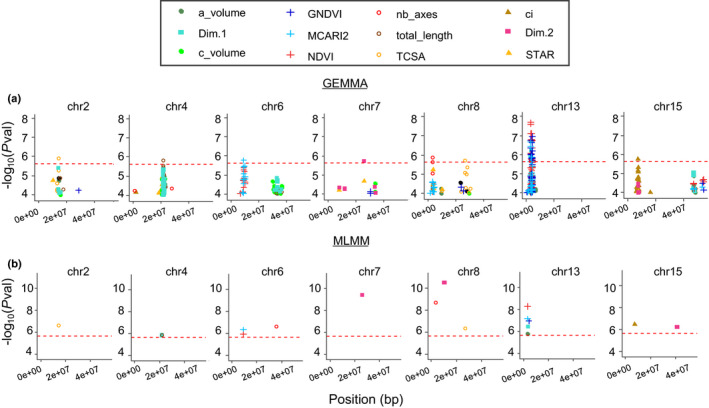
Genome‐wide association studies (GWAS) with single‐SNP and multi‐SNP methods reveal different regions strongly associated with vegetative development‐related traits or light interception‐related traits in an apple tree collection of 241 cultivars. (a) Results from single‐SNP GWAS with gemma. Lodplot for SNPs with −log_10_(*P*val) above 4. (b) Results from multi‐SNP GWAS with MLMM. The red dotted line represents the threshold of −log_10_(*P*val). Only chromosomes with highly reliable SNPs are represented. The complete list of GWAS results is displayed in Supporting Information Table S3. a_volume, alpha hull volume; c_volume, convex hull volume; ci, convexity index; STAR, silhouette to leaf area ratio; total_length, total cumulative axis length; nb_axes, number of axes; TCSA, trunk cross‐sectional area; Dim.1 and Dim.2, respectively first and second principal components of the principal component analysis (PCA) on light interception, plant architecture and shoot traits.

Significant associations were found for all traits related to vegetative architecture, multispectral indices and light interception with a SNP‐by‐SNP model fitted with gemma (Table S3), except for STAR. MLMM reduced the number of significant SNPs in zones where numerous SNPs were significant with gemma and discovered 16 new associations (Table S3). The best model selected by MLMM contained from one to six SNPs depending on the trait. Overall, the proportion of genotypic variation explained by each individual SNP ranged from 2.5% to 14%, while the total proportion jointly explained by the set of SNPs kept by MLMM reached up to 40% (nb_axes and Dim.2, Table S4). We further focused on seven ‘highly reliable’ SNPs, detected with both gemma and MLMM, and on the corresponding genomic regions (Fig. 4; Table 2). For each highly reliable SNP, the count of underlying genes within a ±100 kb interval varied between 12 and 37 and most of these genes were annotated (Tables 2, S5).

**Table 2:** Highly reliable single nucleotide polymorphisms (SNPs) detected for the traits related to vegetative architecture, light interception and canopy temperature response to water deficit computed from terrestrial light detection and ranging (T‐LiDAR), multispectral and thermal imaging on a collection of 241 apple tree cultivars.

Trait	SNP	Chr	Physical. position (bp)	Sign. gemma/sign. MLMM	−log_10_ *P*val gemma	−log_10_ *P*val MLMM	MAF	Allele 1/0	Beta gemma	pve	Mode	Minimum–maximum interval gene.search (bp)	QTL_ID	nb.genes in.interval	nb.genes annotated	Best candidates
TCSA	AX‐115511400	2	14 313 043	Yes/yes	5.9	6.6	0.10	A/C	−0.92	0.09	−0.55	14 213 043–14 413 043	QTL_TCSA_ch2	24	18	One gene involved in vegetative development, one in leaf morphogenesis, one in abscisic acid (ABA) signaling related to growth
resp.TSTA_27.07	AX‐115353652	2	30 732 527	Yes/no	6.2	NA	0.25	C/A	−0.16	0.10	0.46	30 632 527–30 832 527	QTL_resp.TSTA_27.07 _ch2	14	13	One gene epistatic to ABA‐related genes, one Involved in sucrose transport, two involved in ROS response
total_length	AX‐105214995	4	21 791 671	Yes/yes	5.8	5.8	0.18	T/G	−5.24	0.11	0.94	21 691 671–21 891 671	QTL_total_length_ch4	21	19	Three genes related to strigolactones, two to cell wall biosynthesis, one to photomorphogenesis
MCARI2	AX‐115471271	6	9312 975	Yes/yes	5.8	6.3	0.32	T/G	−0.06	0.10	−0.19	9212 975–9412 975	QTL_MCARI2_ch6	15	7	No obvious candidate
Dim.2	AX‐115510439	7	25 727 027	Yes/yes	5.7	9.4	0.08	G/A	0.82	0.06	NA	25 627 027–25 827 027	QTL_Dim.2_ch7	18	14	One gene involved in plant development, two in relation with flowering
resp.TSTA_27.07	AX‐115654802	7	31 053 644	Yes/yes	7.0	7.0	0.05	C/T	0.35	0.12	NA	30 953 644–31 153 644	QTL_resp.TSTA_27.07 _ch7	31	25	One gene involved in ABA‐dependent stress response
nb_axes	AX‐115201917	8	3193 084	Yes/yes	5.9	8.7	0.40	T/C	41.76	0.13	−0.30	3093 084–3293 084	QTL_nb_axes_ch8	19	15	One gene related to gibberelin, one to auxin, two to ABA, one to development
TCSA	AX‐115612404	8	26 695 594	Yes/yes	5.7	6.3	0.16	G/A	−0.77	0.09	−0.44	26 595 594–26 795 594	QTL_TCSA_ch8	12	8	One gene related to cell wall, one to flowering
contrib_17.07	AX‐115355288	12	4961 928	Yes/yes	5.72	5.7	0.12	C/A	0.06	0.08	0.27	4 861 928–5061 928	QTL_contrib_17_07 _ch12	22	19	One gene involved in Ca2+ signalling
MCARI2	AX‐115238182	13	3315 449	Yes/yes	6.7	7.1	0.22	T/G	0.06	0.12	−0.27	3215 449–3415 449	QTL_MCARI2_NDVI _ch13	23	22	One gene involved in PSII, one in chloroplast position, two in shoot development
NDVI	AX‐115238182	13	3315 449	Yes/yes	7.7	8.3	0.22	T/G	0.04	0.13	−0.18	3215 449–3415 449	QTL_MCARI2_NDVI _ch13	23	22	One gene involved in PSII, one in chloroplast position, two in shoot development
a_volume	AX‐115602433	13	3367 459	Yes/yes	5.8	5.8	0.23	C/T	0.10	0.09	0.80	3267 459–3467 459	QTL_a_volume_Dim.1 _ch13	22	21	Two genes involved in shoot development
Dim.1	AX‐115602433	13	3367 459	Yes/yes	6.2	6.5	0.23	C/T	0.54	0.10	0.77	3267 459–3467 459	QTL_a_volume_Dim.1 _ch13	22	21	Two genes involved in shoot development
GNDVI	AX‐115431272	13	4366 451	Yes/yes	6.9	6.9	0.35	G/A	0.02	0.10	0.24	4266 451–4466 451	QTL_GNDVI_ch13	37	37	Two genes involved in chlorophyll degradation
TSTA.WW	AX‐115288925	14	23 753 486	Yes/yes	5.9	5.9	0.48	A/G	0.15	0.09	0.05	23 653 486–23 853 486	QTL_TSTA.WW_ch14	20	14	One channel involved in water transport
ci	AX‐115209148	15	7537 144	Yes/yes	5.8	6.5	0.26	T/C	0.04	0.04	−0.02	7437 144–7637 144	QTL_ci_ch15	31	28	Two genes involved in chlorophyll degradation, one in light signalling, one in photomorphogenesis

Following genome‐wide association studies (GWAS) analyses with a single‐SNP method (gemma) and a multi‐SNP method (MLMM), ‘highly reliable’ SNPs were defined as those significant (‘sign’) with both methods. For each highly reliable SNP, the trait is indicated together with the SNP name, its location on a chromosome and its physical position (in bp, based on the apple genome GDDH13 v.1.1). For each SNP, complementary columns indicate its minor allelic frequency (‘MAF’), the minor and major alleles (allele 0 and 1, respectively), and the allelic effect of the major allele (‘beta’), the percentage of variance explained by the SNP (‘pve’), its mode as retrieved from the single‐SNP analysis, and the ±100 kb interval around the SNP searched for candidate genes. ‘QTL_ID’ is the unique identifier of this interval. The number of genes and the number of annotated genes in this interval are indicated, together with a highlight on the most likely functions identified using both gene ontology and annotation. ‘NA’, not available. Details on GWAS results and the complete list of candidate genes can be found respectively in Supporting Information Tables S3, S5.

Two regions were specifically associated with traits related to light interception: one in the middle of chromosome (chr) 7 (Dim.2) and one at the top of chr15 (ci). The results obtained on the corresponding SNPs for the other traits related to light interception confirmed their substantial, although nonsignificant, effects. For instance, the SNP on chr7 found significant for Dim.2 (−log_10_(*P*val) = 5.73), had a −log_10_(*P*val) of 4.68 for STAR and of 3.84 for ci (Fig. S7a; Table S3). Similarly, the SNP on chr15 significant for ci (−log_10_(*P*val) = 5.77), had a −log_10_(*P*val) of 4.35 for Dim.2 and 3.12 for STAR (Fig. S7b). In both cases, SNPs displayed similar allelic effects for STAR and Dim.2, and opposite effects for ci, consistent with the relationships previously reported between these three traits.

For the variables related to vegetative architecture and multispectral indices, five regions were detected (Figs 4, S8). Three zones were associated with vegetative architecture: (1) one at the top of chr2 (TCSA and Dim.1); (2) one in the middle of chr4 (total_length and Dim.1); and (3) one at the top of chr8 (nb_axes). One zone was specifically associated with MCARI2 and NDVI at the top of chr6. A last zone displaying the highest significance on chr13 was associated with five traits (a_volume, Dim.1, MCARI2, NDVI, GDNVI) with −log_10_(*P*val) up to 7.7 (Table S3). The four first zones earlier‐mentioned (chr2, chr4, chr6 and chr8) were defined either by a single SNP significant for several traits (e.g. on chr2, SNP significant for TCSA and just below the threshold for Dim.1 with parallel allelic effects, Fig. S8a), or by two distinct SNPs in close vicinity and high LD (e.g. on chr6, the SNP detected for MCARI2 and the one detected for NDVI were 28 kb distant with LD = 0.97, Fig. S8c).

**Fig. 5:**
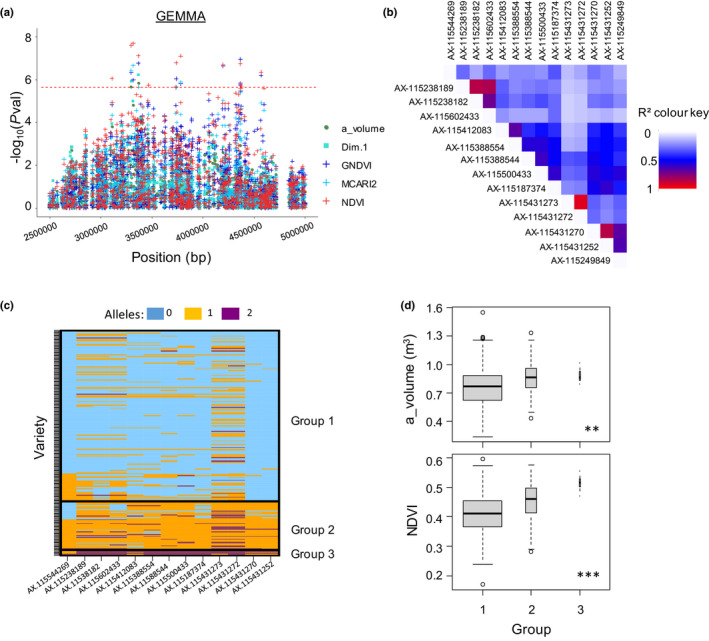
A large genomic region controls architecture‐related traits on chromosome 13 in an apple tree collection of 241 cultivars. (a) Manhattan plots from SNP‐by‐SNP genome‐wide association studies (GWAS) with gemma, focused on chromosome 13 between 2500 and 5000 kb where 14 SNPs were found significant for five vegetative development‐related traits. The red dotted line represents the threshold of Bonferroni (−log_10_(*P*val) = 5.63). (b) Matrix of linkage disequilibrium between the 14 SNPs. (c) Heatmap of allelic values for the 14 SNPs and 241 cultivars ordered based on a hierarchical cluster analysis. The three rectangles (bold, black lines) highlight the three groups from the clustering (respectively group 1, *n* = 183; group 2, *n* = 53; group 3, *n* = 5). (d) Boxplots of genotypic values of a_volume and NDVI per group. Boxplots present the median (centre horizontal line) and interquartile range (grey box) of each group. The upper and lower whiskers represent data within 1.5 × the interquartile range, and values beyond these upper and lower bounds are considered outliers, marked with dots. The significance of the group effect is displayed on the bottom right corner (**, 0.001 < *P* < 0.01; ***, *P* < 0.001). a_volume, alpha hull volume (m^3^); Dim.1, the first principal component coordinate of the principal component analysis (PCA) on light interception and vegetative architecture traits; NDVI, GNDVI and MCARI2, vegetation indices.

The associations found at the top of chr13 corresponded to 14 different SNPs detected with gemma, spanning a distance of 1 Mb (Figs 5a, S9). Although the whole 1 Mb region did not display a particular pattern of high LD (Fig. S10), a hierarchical clustering on the basis of the 14 associated SNPs (Fig. 5c) showed the existence of a first group (183 cultivars) with a majority of homozygous SNPs for the major allele, a second group (53 cultivars) with heterozygous SNPs, and a last one (five cultivars) with homozygous SNPs for the minor allele. This clustering in three groups significantly explained the genotypic variation for both multispectral indices and vegetative architecture‐related traits (Figs 5d, S11). Among the 14 SNPs, three were kept by MLMM, controlling respectively (1) a_volume and Dim.1; (2) NDVI and MCARI2; and (3) GNDVI (Tables 2, S3). The two first SNPs, in high LD (LD > 0.6, Fig. 5b), were located close on the chromosome (52 kb apart, Table S3). Both those SNPS explained 10–14% of the genotypic variation depending on the associated traits and displayed similar MAF (around 0.2). For these two SNPs, the minor allele had a positive effect on the values of the four variables (Fig. S9). This suggests the existence of a common causal polymorphism for a_volume, Dim.1, NDVI and MCARI2. Conversely the SNP significant for GNDVI displayed low LD (Fig. 5b,c) with the two other SNPs and was located further away (1000 kb). This SNP explained 10% of the genotypic variation of GNDVI with a MAF of 0.35. A complementary haplotype‐based analysis performed on this region (Method S1) consistently pointed at two distinct sub‐regions respectively controlling (1) a_volume, Dim.1, NDVI and MCARI2; or (2) GNDVI (Fig. S12).

We tested interactions between SNPs for the traits where several highly reliable SNPs were detected (respectively TCSA and MCARI2 with two SNPs for each, Table 2). No interaction effect between the corresponding SNPs could be detected.

A number of likely candidate genes relevant for vegetative development were identified within the intervals of the highly reliable SNPs (Table S5). The interval surrounding the SNP on chr13 affecting Dim.1 and a_volume contains the gene MD13G1047100 homologous to ABNORMAL SHOOT 5, and MD13G1047200 homologous to BRANCHED 2. Moreover, two genes (MD04G1132000 with two copies and MD04G1132400) homologous to LATERAL BRANCHING OXIDOREDUCTASE 1, were found in the interval for total_length on chr4. In addition, MD08G1043600 homologous to HOMEOBOX GENE 1 and MD08G1043700 homologous to NDL1 were found in the interval for nb_axes on chr8 (Tables 2, S5). By contrast, in the regions specifically associated to vegetation indices, we found genes whose homologous are related to the photosynthetic machinery and chlorophyll content: (1) on chr13, MD13G1046400 homologous to STN7; (2) MD13G1061000, homologous to the pyrimidine reductase PHS1 and (3) MD13G1063900 homologous to the ATNAP transcription activator. We did not identify obvious candidates within the intervals surrounding associations for light interception related traits.

### Genomic regions controlling canopy temperature are independent from those controlling vegetative architecture

**Fig. 6:**
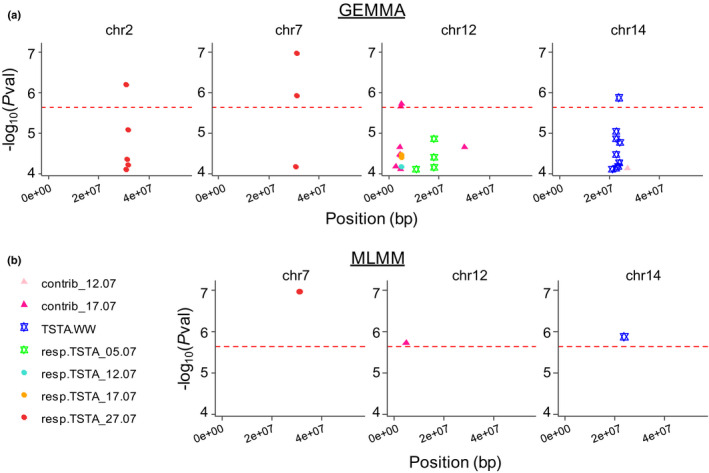
Different genomic regions specifically controlling canopy surface temperature in well‐watered conditions, or in response to water deficit in an apple tree collection of 241 cultivars. (a) Results from single‐SNP genome‐wide association studies (GWAS) with gemma. Lod plot for SNPs with −log_10_(*P*val) above 4. (b) Results from multi‐SNP GWAS with MLMM. The red dotted line represents the threshold of Bonferroni (−log_10_(*P*val) = 5.63). Only chromosomes with significant SNPs are represented. TSTA.WW, canopy surface temperature in well‐watered conditions; resp.TSTA, difference between best linear unbiased predictors (BLUPs) of canopy surface temperature TSTA under water deficit (TSTA.WD) and of TSTA in well‐watered conditions (TSTA.WW), for each of the four measurement dates; contrib_12.07 and contrib_17.07, respectively the ratios resp.TSTA_12.07/resp.TSTA_27.07 and resp.TSTA_17.07/resp.TSTA_27.07.

**Fig. 7:**
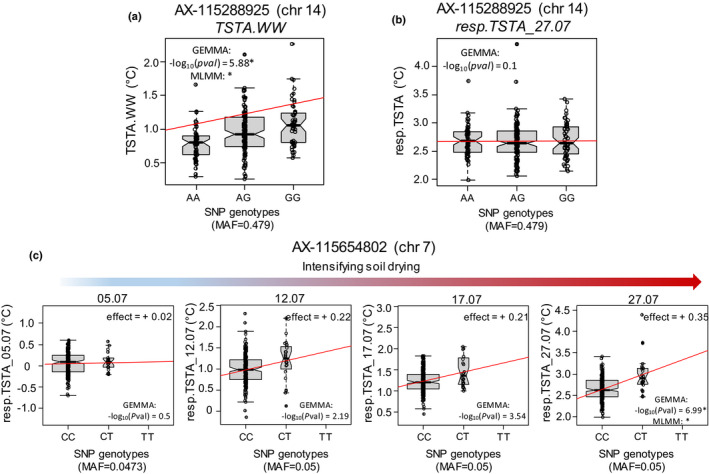
Boxplots of allelic effects for the single nucleotide polymorphisms (SNPs) detected respectively for canopy surface temperature under well‐watered conditions (TSTA.WW), or its response to soil water deficit (resp.TSTA) in an apple tree collection of 241 cultivars. (a) SNP AX‐115288925 on chromosome 14 (chr14) was significantly associated with TSTA.WW with both gemma and MLMM. (b) It had no effect on resp.TSTA. (c) Boxplots of allelic effects at all stages of water deficit, for the SNP on chromosome 7 (chr7) significant for resp.TSTA at the last date (severe water deficit). The −log_10_(*P*val) from SNP‐by‐SNP GWAS with gemma is indicated in the bottom right corner of each boxplot, and a star (*) is added if significant (−log_10_(*P*val) > 5.63). The mention ‘MLMM: *’ is added if the SNP is retained as significant with MLMM. The effect of the minor allele is indicated on the top right corner. MAF, minor allelic frequency. Boxplots present the median (centre horizontal line) and interquartile range (grey box) of each group. The upper and lower whiskers represent data within 1.5 × the interquartile range, and values beyond these upper and lower bounds are considered outliers, marked with dots.

A unique highly reliable SNP controlling TSTA.WW was detected, on chr14 (Fig. 6; Tables 2, S3). It explained 10% of the genotypic variation and displayed homogeneous allele distributions (MAF = 0.48, Fig. 7a). This SNP was close to a gene homologous to the NIP5;1 plasma membrane protein (Table S5). No significant SNP was detected for resp.TSTA on the first three dates. At the last date, i.e. under the most pronounced soil WD, two regions were detected. The first one, located in the middle of chr7 (Fig. 6), was detected both with gemma and MLMM. A second region was detected on chr2 with gemma only. MAF were equal to 0.05 and 0.25 for the SNPs on chr7 and chr2, respectively, and they individually explained 11–12% of the genotypic variance. No interaction effect between those SNPs could be detected with a linear model including all allelic classes at each SNP, probably due to a strong imbalance in the number of observations per combination of genotypic classes. We further investigated whether these SNPs, detected at the most pronounced WD, had some effect at the onset of WD (Fig. 7c). Interestingly, although the SNP on chr7 was not significant on 5, 12 and 17 July, it displayed increasing effects with the intensification of the soil WD. A single highly reliable SNP was detected for contrib_17.07, on chr12 (Fig. 6). No significant SNP was identified for contrib_12.07. Homologous to genes involved in the abscisic acid (ABA) pathway and in the sugar signalling pathway were found in close vicinity to the SNPs on chr2 and chr7: (1) MD07G1239200, homologous to ATAIRP2 TARGET PROTEIN 1; (2) MD02G1255200, homologous to ABCD1, and (3) MD02G1255300 homologous to INV‐E.

Finally, the SNP significant for TSTA.WW had no effect on resp.TSTA (Fig. 7b). Similarly, the two SNPs significant for resp.TSTA had no effect on TSTA.WW (Fig. S13), reinforcing the observation that distinct controls regulate thermal responses of canopy in WW and WD conditions.

## Discussion

### An original combination of high‐throughput field phenotyping with GWAS

To the best of our knowledge, our work is the first to jointly examine genetic variability in tree architecture and canopy functional response to increasing soil drying. The wide genetic background and the original phenotyping tools used allowed us to overcome several bottlenecks encountered by previous studies.

T‐LiDAR measurements provided a fine description of tree architecture, shape, and light interception. Vegetation indices calculated from multispectral imaging proved to be complementary to these descriptors. Finally, estimating canopy surface temperatures from thermal imaging in the same experiment allowed us to assess water‐use strategies on adult trees with complex canopy. Conducting such a study in the field also ensured realistic conditions for root development and soil drying, compared to pot experiments (Turner, 2019).

For all traits, high genetic coefficients of variation indicated diversified adaptive strategies in the core‐collection. This core‐collection mostly consists of French but also European old dessert apple varieties with a large genetic variability due to the heterozygous nature of domesticated apple and the large gene flow at the European scale as shown by Urrestarazu *et al*. (2016).

We jointly used two GWAS methods, gemma (Zhou & Stephens, 2012) and MLMM (Segura *et al*., 2012), and defined ‘highly reliable’ associations for further in‐depth analysis, which corresponded to SNPs that were significant for the same trait with both methods. These associations were also often just below the threshold of significance for other highly related traits (e.g. STAR, ci and Dim.2 on chr7 and chr15). With this approach, a single or two highly reliable genomic regions were detected for each trait with an individual SNP effect explaining 5–15% of the genotypic variation, despite the fairly high values of genomic heritability estimated for all traits (around 0.5). Taken together, these results confirm that these architectural traits are controlled by polygenic effects of low intensity distributed along the genome. Epistasis interactions could also influence the phenotypic variations. However, the size of the population yielding low MAF values for certain markers hampered the possibilities to detect such interactions. Extending such studies to a broader genetic background (Jung *et al*., 2020) could unravel these interactions.

### Architectural traits and vegetation indices are controlled by common and specific genomic regions

We characterized tree architecture through many different traits (Fig. 1). PCA allowed to transform this massive set of correlated traits into a reduced set of two principal component (PC) coordinates (He *et al*., 2008). This strategy increased detection power, as GWAS on these integrated variables revealed different causal genomic regions for Dim.1 (vegetative development) and for Dim.2 (light interception capacity), and the discovery of genomic regions that had been overlooked for individual traits (e.g. association for Dim.2 on chr7).

Five highly reliable SNPs were associated with vegetative architecture (Table 2), for TCSA (chr2 and chr8), total_length (chr4), nb_axes (chr8), and for Dim.1 and a_volume (chr13). Two of these regions co‐localized with QTLs previously reported in biparental progenies on related traits (Table S6). The SNP found for total_length on chr4 echoes the localization of a QTL for the number of axillary shoots at tree scale, and is in close vicinity to a QTL for mean internode length on proleptic shoot found by Segura *et al*. (2009). These traits are very likely to contribute to the total cumulative axis length considered here, reinforcing the association reported in the present study. Conversely, the new regions discovered in our study on chr2 and chr8 were not previously reported, emphasizing the interest of using a broad genetic diversity.

The SNPs detected for vegetative development pointed to highly relevant candidate genes. In particular we identified in the intervals genes homologous to (1) ABNORMAL SHOOT 5, that was shown to have a crucial role for leaf development and morphogenesis (An *et al*., 2014; chr13); (2) BRANCHED 2 that controls axillary bud development (Aguilar‐Martínez *et al*., 2007; chr13); (3) LATERAL BRANCHING OXIDOREDUCTASE 1, involved in the biosynthesis of strigolactones (total_length on chr4). Strigolactones are known to suppress bud outgrowth (Gomez‐Roldan *et al*., 2008) and this function was shown to be conserved in trees such as *Populus* (Muhr *et al*., 2016).

Genes related to two other hormones involved in shoot development were also identified in close vicinity of the SNP detected on chr8 for nb_axes. These genes are respectively homologous to HOMEOBOX GENE 1 and to NDL1 that both contribute to the control of zonation and lateral organ outgrowth in apical meristems via gibberellin biosynthesis (Gómez‐Mena & Sablowski, 2008), and auxin transport (Mudgil *et al*., 2013), respectively.

It is striking that specific genomic regions were detected for vegetation indices (NDVI, MCARI2, GNDVI) distinct from those associated with vegetative development, highlighting the complementary nature of the information they carry. These associations were consistent with the chlorophyll‐related characteristics often attributed to vegetation indices. For instance, on chr13, the gene homologous to STN7, a protein kinase involved in photosynthesis regulation was found in the region specifically associated to MCARI2 and NDVI. This kinase plays a well‐described role in the redistribution of light excitation energy between photosystem II and photosystem I (Depège *et al*., 2003; Lemeille *et al*., 2009). It is noteworthy that GNDVI that has been proposed to display more sensitivity to chlorophyll concentration than the two other indices (Gitelson *et al*., 1996), revealed complementary regions. Here indeed, two genes located in the interval associated with GNDVI only were associated with chlorophyll degradation: the pyrimidine reductase PHS1 (Ouyang *et al*., 2010) and the transcription activator NAP (Lei *et al*., 2020).

### A large variation in water economy strategy under genetic control

Although water deficit is a major limiting factor for plant growth and production, its impact on genetic variability in fruit trees has mostly been studied on young potted plants (Lauri *et al*., 2011), or detached leaves (Bassett *et al*., 2015). Here we deployed a more realistic approach in the orchard, by withholding irrigation over one month in summer resulting in a gradual water limitation that echoes the tense situations on the water resource typically encountered in the Mediterranean regions (Sofo *et al*., 2012). Because the water deficit was short and late during the tree growth season, architecture related traits were not, or only slightly affected by WD, contrarily to the functional traits.

In this study, water potential values gradually differentiated between WW and WD trees. At the last date of measurements, mean *ψ*_stem,midday_ reached −1.8 MPa for WD trees, representative of a well‐established water deficit (Naor, 2006).

It is noteworthy that the difference between canopy and air temperature (TSTA) considered as a proxy of canopy transpiration (Jones *et al*., 2009) revealed strong diversity among varieties in the WW scenario (Fig. S4). A highly reliable SNP was found on chr14 for TSTA.WW, close to NIP5;1 that belongs to NIPs aquaporin family with a water channel activity (Takano *et al*., 2006). Aquaporin activity in leaves has been correlated with leaf hydraulic conductance in perennial species such as grapevine (Pou *et al*., 2013). Because close feedbacks link water transport and stomatal opening (Sack & Holbrook, 2006), this could suggest that variations in constitutive water transport might play an important role in the genetic variability in canopy temperature in WW conditions.

The response of canopy surface temperature to soil water deficit (resp.TSTA) increased with its severity, and a strong and increasing genotypic variability was observed along the month of measurements. It should be noted that in such an orchard experiment, the evolution of the soil WD was only partly controlled, resulting in variability for *Ψ*_soil_ (Fig. S1) as well as *Ψ*_leaf,predawn_ (Table S2) at any date of measurements. Genetic variations observed might thus arise from a genotypic capacity to limit soil water depletion, rather than only from a genotypic response to the same WD level and despite all trees growing on the same rootstock. The variations in resp.TSTA observed within the collection likely reflect variability in stomatal closure, as suggested by the genes underlying the corresponding associations, involved in the widely conserved ABA and sugar signalling pathways. Two genes involved in the ABA pathway (ATAIRP2 TARGET PROTEIN 1 that mediates ABA‐dependent stress response (Oh *et al*., 2017), and ABCD1 which is epistatic to ABI3 and ABA1 that both affect ABA signalling in guard cells (Parcy & Giraudat, 1997)) were found in the associated intervals on chr2 and chr7. Genes of the sugar signalling pathway were revealed such as INV‐E involved in starch and sucrose metabolic process (Vargas *et al*., 2008). Sugars, as end products of photosynthesis, contribute to stomatal closure of various angiosperm species to coordinate sugar production with water loss (Kottapalli *et al*., 2018). Strikingly, although these SNPs were only significant at the strongest soil dryness intensity (resp.TSTA.27_07), they showed consistent and increasing effects at lighter WD intensities (Fig. 7c). This suggests that the corresponding signalling pathways may also play a role at light and moderate WD. Variation in the ranking of cultivars by WD intensity (Fig. 2d) as well as the relatively weak SNP effects (Table S3) detected for resp.TSTA and contrib_17.07 are consistent with the many genes known to control stomatal differentiation (Bertolino *et al*., 2019) and function (Ward *et al*., 2009), with a variable contribution depending on WD intensity.

### Independent controls of tree architecture and canopy surface temperature response to soil drying

A major outcome of this study is that phenotypic and genetic analyses indicate independent controls of traits respectively related to (1) vegetative architecture; (2) light interception; and (3) water loss estimated by canopy surface temperature. This represents an important step in our understanding of the physiological and genetic basis of these three groups of traits, which are all relevant breeding targets in the context of climate change. As a consequence, multiple allelic combinations exist within the apple tree collection for the 16 highly reliable SNPs (Table S7; Fig. S14). This allows exploring for specific combinations. For instance, results presented in Table S7 and Fig. S14 indicate that a large subset of cultivars bear alleles favourable to maximizing light interception efficiency, which could be an advantageous feature in the view of maximizing the production of photo‐assimilates at the tree scale. Within this subgroup, cultivars could then be targeted with alleles conferring either high or low increase in canopy surface temperature under water deficit, indicative of more or less stomatal closure. While stomatal closure may be beneficial to save water and avoid catastrophic drops in leaf water potential, stomatal opening may on the contrary be beneficial to carbon assimilation and leaf cooling. The likelihood of negative or positive outcomes of each allelic combination will strongly depend on the severity of water deficit events encountered during the growing seasons (Tardieu, 2012). These results open promising avenues, suggesting that breeding schemes could incorporate genotypes carrying favourable alleles for all three groups of architectural and functional traits. Importantly, there is no unique ideal combination of architectural and functional traits. Ideotypes shall be reasoned depending on environmental constraints and production objectives.

## Author contributions

AC‐L, BP, MD, J‐LR, and EC designed research. AC‐L, BP, MD, and J‐LR performed the experiments. MD performed the MS and TIR images post‐processing. BP performed the T‐LiDAR scans post‐processing. AC‐L and BP analysed data and carried out GWAS analyses with contribution from VS and BG. HM and C‐ED generated the genotyping data. AC‐L and BP wrote the article with the contribution of EC, J‐LR, C‐ED, HM, VS and MD. All authors approved the manuscript.

## Supporting information


**Fig. S1** Evolution of soil water potential during the experiment.
**Fig. S2** Tree architecture and light‐interception related traits retrieved from T‐LiDAR measurements.
**Fig. S3** Vegetation indices (NDVI, GNDVI and MCARI2) are stable and correlated across dates and watering scenarios.
**Fig. S4** Canopy surface temperature (TSTA) under the well‐watered scenario across dates.
**Fig. S5** Correlations between the proportion of maximal canopy temperature increase observed at the light and moderate intensities of soil dryness, and the maximal canopy temperature increase.
**Fig. S6** Weak correlations between tree canopy temperature and traits related to vegetative architecture and light interception.
**Fig. S7** Two regions associated with traits related to light interception capacity on chromosomes 7 and 15.
**Fig. S8** Three regions associated with traits related to tree architecture on chromosomes 2, 4, and 6.
**Fig. S9** Boxplots of allelic effects for a large region associated with several architecture‐related traits on chromosome 13.
**Fig. S10** Heatmap of linkage disequilibrium in a large region associated with several architecture‐related traits on chromosome 13.
**Fig. S11** A large genomic region controls architecture‐related traits on chromosome 13.
**Fig. S12** Haplotype analysis of a genomic region controlling architecture‐related traits on chromosome 13.
**Fig. S13** Different regions controlling respectively the canopy temperature in well‐watered conditions, or its response to water deficit.
**Fig. S14** Heatmap of allelic effects for the 16 highly reliable associations and the 241 cultivars of the apple tree core‐collection.
**Methods S1** Haplotype analysis of a genomic region controlling architecture‐related traits on chromosome 13.
**Table S1** Summary of the meteorological variables during the drones’ flights at the four dates of measurements in July 2017.
**Table S2** Leaf and stem water potentials measured on 45 trees among the apple tree core‐collection.

**Table S3** Complete set of associations detected for the traits related to vigour, light interception and canopy temperature response to water deficit computed from T‐LiDAR, multispectral and thermal imaging on a collection of 241 apple tree varieties.
**Table S4** Summary of the GWAS results per trait.
**Table S5** List of genes underlying the most highly reliable SNPs.
**Table S6** Co‐localizations between associated SNPs on the apple core collection and SSR associated on bi‐parental populations for similar or related traits.
**Table S7** List of allelic effects for the 16 highly reliable associations and the 241 cultivars of the apple tree core‐collection.
Please note: Wiley Blackwell are not responsible for the content or functionality of any Supporting Information supplied by the authors. Any queries (other than missing material) should be directed to the *New Phytologist* Central Office.

## Data Availability

Raw data and BLUPs of phenotypes together with the list of the 241 cultivars with the recently attributed MUNQ codes (for Malus UNiQue genotype code, Denancé *et al*., 2020) are publicly available in Coupel‐Ledru *et al*. (2022) at this site: https://doi.org/10.15454/C8IPII. Genotyping data of the collection used in this study are publicly available at this site: https://doi.org/10.15454/F5XIVJ (Denancé *et al*., 2022). Additional relevant data are within the manuscript and its Supporting Information files.

## References

[nph17960-bib-0001] Aguilar‐Martínez JA , Poza‐Carrión C , Cubas P . 2007. Arabidopsis BRANCHED1 acts as an integrator of branching signals within axillary buds. Plant Cell 19: 458–472. 17307924 10.1105/tpc.106.048934 PMC1867329

[nph17960-bib-0002] Alexander DH , Lange K . 2011. Enhancements to the ADMIXTURE algorithm for individual ancestry estimation. BMC Bioinformatics 12: 246. 21682921 10.1186/1471-2105-12-246 PMC3146885

[nph17960-bib-0003] An R , Liu X , Wang R , Wu H , Liang S , Shao J , Qi Y , An L , Yu F . 2014. The over‐expression of two transcription factors, ABS5/bHLH30 and ABS7/MYB101, leads to upwardly curly leaves. PLoS ONE 9: e107637. 25268707 10.1371/journal.pone.0107637 PMC4182325

[nph17960-bib-0004] Araus JL , Cairns JE . 2014. Field high‐throughput phenotyping: the new crop breeding frontier. Trends in Plant Science 19: 52–61. 24139902 10.1016/j.tplants.2013.09.008

[nph17960-bib-0005] Barthélémy D , Caraglio Y . 2007. Plant architecture: a dynamic, multilevel and comprehensive approach to plant form, structure and ontogeny. Annals of Botany 99: 375–407. 17218346 10.1093/aob/mcl260 PMC2802949

[nph17960-bib-0006] Bassett C , Artlip T , Wisniewski M. 2015. Water deficit treatment and measurement in apple trees. BIO‐PROTOCOL 5. 10.21769/bioprotoc.1388

[nph17960-bib-0007] Belaj A , Dominguez‐García MDC , Atienza SG , Martín Urdíroz N , De la Rosa R , Satovic Z , Martín A , Kilian A , Trujillo I , Valpuesta V *et al*. 2012. Developing a core collection of olive (*Olea europaea* L.) based on molecular markers (DArTs, SSRs, SNPs) and agronomic traits. Tree Genetics & Genomes 8: 365–378.

[nph17960-bib-0008] Bertheloot J , Barbier F , Boudon F , Perez‐Garcia MD , Péron T , Citerne S , Dun E , Beveridge C , Godin C , Sakr S . 2020. Sugar availability suppresses the auxin‐induced strigolactone pathway to promote bud outgrowth. New Phytologist 225: 866–879. 31529696 10.1111/nph.16201

[nph17960-bib-0009] Bertolino LT , Caine RS , Gray JE . 2019. Impact of stomatal density and morphology on water‐use efficiency in a changing world. Frontiers in Plant Science 10: 225. 30894867 10.3389/fpls.2019.00225 PMC6414756

[nph17960-bib-0010] Bianco L , Cestaro A , Linsmith G , Muranty H , Denancé C , Théron A , Poncet C , Micheletti D , Kerschbamer E , Pierro EAD *et al*. 2016. Development and validation of the Axiom®Apple480K SNP genotyping array. The Plant Journal 86: 62–74. 26919684 10.1111/tpj.13145

[nph17960-bib-0011] Boudon F , Preuksakarn C , Ferraro P , Diener J , Nacry P , Nikinmaa E , Godin C . 2014. Quantitative assessment of automatic reconstructions of branching systems obtained from laser scanning. Annals of Botany 114: 853–862. 24769534 10.1093/aob/mcu062 PMC4156121

[nph17960-bib-0012] Costes E , Gion J‐M . 2015. Chapter five – genetics and genomics of tree architecture. In: Plomion C , Adam‐Blondon A‐F , eds. Land plants – trees. Advances in botanical research. New York, NY, USA: Academic Press, 157–200.

[nph17960-bib-0013] Coupel‐Ledru A , Lebon É , Christophe A , Doligez A , Cabrera‐Bosquet L , Péchier P , Hamard P , This P , Simonneau T . 2014. Genetic variation in a grapevine progeny (*Vitis vinifera* L. cvs Grenache×Syrah) reveals inconsistencies between maintenance of daytime leaf water potential and response of transpiration rate under drought. Journal of Experimental Botany 65: 6205–6218. 25381432 10.1093/jxb/eru228 PMC4223985

[nph17960-bib-0014] Coupel‐Ledru A , Pallas B , Delalande M , Boudon F , Carrié E , Martinez S , Regnard J‐L , Costes E . 2019. Multi‐scale high‐throughput phenotyping of apple architectural and functional traits in orchard reveals genotypic variability under contrasted watering regimes. Horticulture Research 6: 1–15. 31044079 10.1038/s41438-019-0137-3 PMC6491481

[nph17960-bib-0015] Coupel‐Ledru A , Pallas B , Delalande M , Boudon F , Carrié E , Martinez S , Regnard J‐L , Costes E . 2022. Tree architecture, canopy shape, vegetation indices and canopy surface temperature related traits in response to soil drying in an apple tree core‐collection. *Portail Data INRAE, V1*. Dataset data.inrae.fr. doi: 10.15454/C8IPII [accessed 1 December 2021].

[nph17960-bib-0016] Daccord N , Celton J‐M , Linsmith G , Becker C , Choisne N , Schijlen E , van de Geest H , Bianco L , Micheletti D , Velasco R *et al*. 2017. High‐quality de novo assembly of the apple genome and methylome dynamics of early fruit development. Nature Genetics 49: 1099–1106. 28581499 10.1038/ng.3886

[nph17960-bib-0017] Dardick C , Callahan A , Horn R , Ruiz KB , Zhebentyayeva T , Hollender C , Whitaker M , Abbott A , Scorza R . 2013. PpeTAC1 promotes the horizontal growth of branches in peach trees and is a member of a functionally conserved gene family found in diverse plants species. The Plant Journal 75: 618–630. 23663106 10.1111/tpj.12234

[nph17960-bib-0018] Denancé C , Muranty H , Durel C‐E . 2020. MUNQ – Malus UNiQue genotype code for grouping apple accessions corresponding to a unique genotypic profile. *Portail Data INRAE, V1*. Dataset data.inrae.fr. doi: 10.15454/HKGMAS [accessed 1 December 2021].

[nph17960-bib-0019] Denancé C , Muranty H , Durel C‐E . 2022. FruitBreedomics apple 275K SNP genotypic data. *Portail Data INRAE, V1*. Dataset data.inrae.fr. doi: 10.15454/F5XIVJ [accessed 1 December 2021].

[nph17960-bib-0020] Depège N , Bellafiore S , Rochaix J‐D . 2003. Role of chloroplast protein kinase Stt7 in LHCII phosphorylation and state transition in *Chlamydomonas* . Science 299: 1572–1575. 12624266 10.1126/science.1081397

[nph17960-bib-0021] Flutre T . 2019. Timothee Flutre’s personal R code. v.0.170.0. [WWW document] URL https://github.com/timflutre/rutilstimflutre [accessed 1 June 2021].

[nph17960-bib-0022] Fournier M , Dlouhá J , Jaouen G , Almeras T . 2013. Integrative biomechanics for tree ecology: beyond wood density and strength. Journal of Experimental Botany 64: 4793–4815. 24014867 10.1093/jxb/ert279

[nph17960-bib-0023] Franks PJ , Drake PL , Froend RH . 2007. Anisohydric but isohydrodynamic: seasonally constant plant water potential gradient explained by a stomatal control mechanism incorporating variable plant hydraulic conductance. Plant, Cell & Environment 30: 19–30. 10.1111/j.1365-3040.2006.01600.x 17177873

[nph17960-bib-0024] Garcia‐Lor A , Luro F , Ollitrault P , Navarro L . 2017. Comparative analysis of core collection sampling methods for mandarin germplasm based on molecular and phenotypic data. Annals of Applied Biology 171: 327–339.

[nph17960-bib-0025] Gitelson AA , Kaufman YJ , Merzlyak MN . 1996. Use of a green channel in remote sensing of global vegetation from EOS‐MODIS. Remote Sensing of Environment 58: 289–298.

[nph17960-bib-0026] Gómez‐Mena C , Sablowski R . 2008. ARABIDOPSIS THALIANA HOMEOBOX GENE1 establishes the basal boundaries of shoot organs and controls stem growth. Plant Cell 20: 2059–2072. 18757555 10.1105/tpc.108.059188 PMC2553610

[nph17960-bib-0027] Gomez‐Roldan V , Fermas S , Brewer PB , Puech‐Pagès V , Dun EA , Pillot J‐P , Letisse F , Matusova R , Danoun S , Portais J‐C *et al*. 2008. Strigolactone inhibition of shoot branching. Nature 455: 189–194. 18690209 10.1038/nature07271

[nph17960-bib-0028] Haboudane D , Miller JR , Pattey E , Zarco‐Tejada PJ , Strachan IB . 2004. Hyperspectral vegetation indices and novel algorithms for predicting green LAI of crop canopies: modeling and validation in the context of precision agriculture. Remote Sensing of Environment 90: 337–352.

[nph17960-bib-0029] He L‐N , Liu Y‐J , Xiao P , Zhang L , Guo Y , Yang T‐L , Zhao L‐J , Drees B , Hamilton J , Deng H‐Y *et al*. 2008. Genomewide linkage scan for combined obesity phenotypes using principal component analysis. Annals of Human Genetics 72: 319–326. 18261184 10.1111/j.1469-1809.2007.00423.x

[nph17960-bib-0030] Hollender CA , Dardick C . 2015. Molecular basis of angiosperm tree architecture. New Phytologist 206: 541–556. 25483362 10.1111/nph.13204

[nph17960-bib-0031] Jones HG , Serraj R , Loveys BR , Xiong L , Wheaton A , Price AH . 2009. Thermal infrared imaging of crop canopies for the remote diagnosis and quantification of plant responses to water stress in the field. Functional Plant Biology 36: 978–989. 32688709 10.1071/FP09123

[nph17960-bib-0032] Jung M , Roth M , Aranzana MJ , Auwerkerken A , Bink M , Denancé C , Dujak C , Durel C‐E , Font i Forcada C , Cantin CM *et al*. 2020. The apple REFPOP—a reference population for genomics‐assisted breeding in apple. Horticulture Research 7: 1–16. 33328447 10.1038/s41438-020-00408-8 PMC7603508

[nph17960-bib-0033] Kenis K , Keulemans J . 2007. Study of tree architecture of apple (*Malus × domestica* Borkh.) by QTL analysis of growth traits. Molecular Breeding 19: 193–208.

[nph17960-bib-0034] Klein T . 2014. The variability of stomatal sensitivity to leaf water potential across tree species indicates a continuum between isohydric and anisohydric behaviours. Functional Ecology 28: 1313–1320.

[nph17960-bib-0035] Kottapalli J , David‐Schwartz R , Khamaisi B , Brandsma D , Lugassi N , Egbaria A , Kelly G , Granot D . 2018. Sucrose‐induced stomatal closure is conserved across evolution. PLoS ONE 13: e0205359. 30312346 10.1371/journal.pone.0205359 PMC6185732

[nph17960-bib-0036] Lassois L , Denancé C , Ravon E , Guyader A , Guisnel R , Hibrand‐Saint‐Oyant L , Poncet C , Lasserre‐Zuber P , Feugey L , Durel C‐E . 2016. Genetic diversity, population structure, parentage analysis, and construction of core collections in the French apple germplasm based on SSR markers. Plant Molecular Biology Reporter 34: 827–844.

[nph17960-bib-0037] Lauri P‐É , Barigah TS , Lopez G , Martinez S , Losciale P , Zibordi M , Manfrini L , Corelli‐Grappadelli L , Costes E , Regnard J‐L . 2016. Genetic variability and phenotypic plasticity of apple morphological responses to soil water restriction in relation with leaf functions and stem xylem conductivity. Trees 30: 1893–1908.

[nph17960-bib-0038] Lauri P‐É , Gorza O , Cochard H , Martinez S , Celton J‐M , Ripetti V , Lartaud M , Bry X , Trottier C , Costes E . 2011. Genetic determinism of anatomical and hydraulic traits within an apple progeny. Plant, Cell & Environment 34: 1276–1290. 10.1111/j.1365-3040.2011.02328.x 21477120

[nph17960-bib-0039] Lawson T , Blatt MR . 2014. Stomatal size, speed, and responsiveness impact on photosynthesis and water use efficiency. Plant Physiology 164: 1556–1570. 24578506 10.1104/pp.114.237107 PMC3982722

[nph17960-bib-0040] Lei W , Li Y , Yao X , Qiao K , Wei L , Liu B , Zhang D , Lin H . 2020. NAP is involved in GA‐mediated chlorophyll degradation and leaf senescence by interacting with DELLAs in Arabidopsis. Plant Cell Reports 39: 75–87. 31646371 10.1007/s00299-019-02474-2

[nph17960-bib-0041] Lemeille S , Willig A , Depège‐Fargeix N , Delessert C , Bassi R , Rochaix J‐D . 2009. Analysis of the chloroplast protein kinase Stt7 during state transitions. PLoS Biology 7: e1000045. 10.1371/journal.pbio.1000045 PMC2650728 19260761

[nph17960-bib-0042] Lines ER , Zavala MA , Purves DW , Coomes DA . 2012. Predictable changes in aboveground allometry of trees along gradients of temperature, aridity and competition. Global Ecology and Biogeography 21: 1017–1028.

[nph17960-bib-0043] Liu BH , Cheng L , Liang D , Zou YJ , Ma FW . 2012. Growth, gas exchange, water‐use efficiency, and carbon isotope composition of ‘Gale Gala’ apple trees grafted onto 9 wild Chinese rootstocks in response to drought stress. Photosynthetica 50: 401–410.

[nph17960-bib-0044] Massonnet C , Regnard JL , Lauri PÉ , Costes E , Sinoquet H . 2008. Contributions of foliage distribution and leaf functions to light interception, transpiration and photosynthetic capacities in two apple cultivars at branch and tree scales. Tree Physiology 28: 665–678. 18316299 10.1093/treephys/28.5.665

[nph17960-bib-0045] Monteith JL . 1965. Evaporation and environment. In: Fogg GE , ed. Symposium of the society for experimental biology, the state and movement of water in living organisms. New York: Academic Press, 205–234.

[nph17960-bib-0046] Mudgil Y , Ghawana S , Jones AM . 2013. N‐MYC DOWN‐REGULATED‐LIKE proteins regulate meristem initiation by modulating auxin transport and MAX2 expression. PLoS ONE 8: e77863. 24223735 10.1371/journal.pone.0077863 PMC3817199

[nph17960-bib-0047] Muhr M , Prüfer N , Paulat M , Teichmann T . 2016. Knockdown of strigolactone biosynthesis genes in *Populus* affects BRANCHED1 expression and shoot architecture. New Phytologist 212: 613–626. 27376674 10.1111/nph.14076

[nph17960-bib-0048] Naor A . 2006. Irrigation scheduling and evaluation of tree water ttatus in deciduous orchards. In: Janick J , ed. Horticultural reviews. Hoboken, NJ, USA: John Wiley & Sons, 111–165.

[nph17960-bib-0049] Naor A , Naschitz S , Peres M , Gal Y . 2008. Responses of apple fruit size to tree water status and crop load. Tree Physiology 28: 1255–1261. 18519256 10.1093/treephys/28.8.1255

[nph17960-bib-0050] Ngao J , Martinez S , Marquier A , Bluy S , Saint‐Joanis B , Costes E , Pallas B . 2020. Spatial variability in carbon and nitrogen related traits in apple trees: the effects of the light environment and crop load. Journal of Experimental Botany 72: 1933–1945. 10.1093/jxb/eraa559 33249486

[nph17960-bib-0051] Oh TR , Kim JH , Cho SK , Ryu MY , Yang SW , Kim WT . 2017. AtAIRP2 E3 ligase affects ABA and high‐salinity responses by stimulating its ATP1/SDIRIP1 substrate turnover. Plant Physiology 174: 2515–2531. 28626006 10.1104/pp.17.00467 PMC5543955

[nph17960-bib-0052] Ouyang M , Ma J , Zou M , Guo J , Wang L , Lu C , Zhang L . 2010. The photosensitive phs1 mutant is impaired in the riboflavin biogenesis pathway. Journal of Plant Physiology 167: 1466–1476. 20580123 10.1016/j.jplph.2010.05.005

[nph17960-bib-0053] Pallas B , Martinez S , Simler O , Carrié E , Costes E , Boudon F . 2020. Assessing T‐LiDAR technology for high throughput phenotyping apple tree topological and architectural traits. Acta Horticulturae 1281: 625–632.

[nph17960-bib-0054] Parcy F , Giraudat J . 1997. Interactions between the ABI1 and the ectopically expressed ABI3 genes in controlling abscisic acid responses in Arabidopsis vegetative tissues. The Plant Journal 11: 693–702. 9161030 10.1046/j.1365-313x.1997.11040693.x

[nph17960-bib-0055] Pou A , Medrano H , Flexas J , Tyerman SD . 2013. A putative role for TIP and PIP aquaporins in dynamics of leaf hydraulic and stomatal conductances in grapevine under water stress and re‐watering: grapevine leaf conductances and aquaporins. Plant, Cell & Environment 36: 828–843. 10.1111/pce.12019 23046275

[nph17960-bib-0056] Pradal C , Boudon F , Nouguier C , Chopard J , Godin C . 2009. PlantGL: a Python‐based geometric library for 3D plant modelling at different scales. Graphical Models 71: 1–21.

[nph17960-bib-0057] Pradal C , Dufour‐Kowalski S , Boudon F , Fournier C , Godin C . 2008. OpenAlea: a visual programming and component‐based software platform for plant modelling. Functional Plant Biology 35: 751–760. 32688829 10.1071/FP08084

[nph17960-bib-0058] Prunier J , Pelgas B , Gagnon F , Desponts M , Isabel N , Beaulieu J , Bousquet J . 2013. The genomic architecture and association genetics of adaptive characters using a candidate SNP approach in boreal black spruce. BMC Genomics 14: 368. 23724860 10.1186/1471-2164-14-368 PMC3674900

[nph17960-bib-0059] Purcell S , Neale B , Todd‐Brown K , Thomas L , Ferreira MAR , Bender D , Maller J , Sklar P , de Bakker PIW , Daly MJ *et al*. 2007. Plink: a tool set for whole‐genome association and population‐based linkage analyses. American Journal of Human Genetics 81: 559–575. 17701901 10.1086/519795 PMC1950838

[nph17960-bib-0060] R Development Core Team . 2020. R: a language and environment for statistical computing. Vienna, Austria: R Foundation for Statistical Computing.

[nph17960-bib-0061] Rebolledo MC , Luquet D , Courtois B , Henry A , Soulié J‐C , Rouan L , Dingkuhn M . 2013. Can early vigour occur in combination with drought tolerance and efficient water use in rice genotypes? Functional Plant Biology 40: 582–594. 32481132 10.1071/FP12312

[nph17960-bib-0062] Rouse JW . 1974. Monitoring the vernal advancement and retrogradation (green wave effect) of natural vegetation. Third ERTS Symposium 1: NASA SP‐351: 309–317.

[nph17960-bib-0063] Sack L , Holbrook NM . 2006. Leaf hydraulics. Annual Review of Plant Biology 57: 361–381. 10.1146/annurev.arplant.56.032604.144141 16669766

[nph17960-bib-0064] Sade N , Gebremedhin A , Moshelion M . 2012. Risk‐taking plants: anisohydric behavior as a stress‐resistance trait. Plant Signaling & Behavior 7: 767–770. 22751307 10.4161/psb.20505 PMC3583960

[nph17960-bib-0065] Santini F , Kefauver SC , Araus JL , de Dios VR , García SM , Grivet D , Voltas J . 2021. Bridging the genotype–phenotype gap for a Mediterranean pine by semi‐automatic crown identification and multispectral imagery. New Phytologist 229: 245–258. 32893885 10.1111/nph.16862

[nph17960-bib-0066] Schultz HR . 2003. Differences in hydraulic architecture account for near‐isohydric and anisohydric behaviour of two field‐grown *Vitis vinifera* L. cultivars during drought. Plant, Cell & Environment 26: 1393–1405.

[nph17960-bib-0067] Segura V , Cilas C , Costes E . 2008. Dissecting apple tree architecture into genetic, ontogenetic and environmental effects: mixed linear modelling of repeated spatial and temporal measures. New Phytologist 178: 302–314. 18248585 10.1111/j.1469-8137.2007.02374.x

[nph17960-bib-0068] Segura V , Denancé C , Durel C‐E , Costes E . 2007. Wide range QTL analysis for complex architectural traits in a 1‐year‐old apple progeny. Genome 50: 159–171. 17546081 10.1139/g07-002

[nph17960-bib-0069] Segura V , Durel C‐E , Costes E . 2009. Dissecting apple tree architecture into genetic, ontogenetic and environmental effects: qTL mapping. Tree Genetics & Genomes 5: 165–179. 10.1111/j.1469-8137.2007.02374.x 18248585

[nph17960-bib-0070] Segura V , Vilhjálmsson BJ , Platt A , Korte A , Seren Ü , Long Q , Nordborg M . 2012. An efficient multi‐locus mixed‐model approach for genome‐wide association studies in structured populations. Nature Genetics 44: 825–830. 22706313 10.1038/ng.2314 PMC3386481

[nph17960-bib-0071] Sinoquet H , Roux XL , Adam B , Ameglio T , Daudet FA . 2001. RATP: a model for simulating the spatial distribution of radiation absorption, transpiration and photosynthesis within canopies: application to an isolated tree crown. Plant, Cell & Environment 24: 395–406.

[nph17960-bib-0072] Sofo A , Palese AM , Casacchia T , Dichio B , Xiloyannis C . 2012. Sustainable fruit production in Mediterranean orchards subjected to drought stress. In: Ahmad P , Prasad MNV , eds. Abiotic stress responses in plants: metabolism, productivity and sustainability. New York, NY, USA: Springer, 105–129.

[nph17960-bib-0073] Souza LM , Gazaffi R , Mantello CC , Silva CC , Garcia D , Guen VL , Cardoso SEA , Garcia AAF , Souza AP . 2013. QTL mapping of growth‐related traits in a full‐sib family of rubber tree (*Hevea brasiliensis*) evaluated in a sub‐tropical climate. PLoS ONE 8: e61238. 23620732 10.1371/journal.pone.0061238 PMC3631230

[nph17960-bib-0074] Strong D , Azarenko AN . 2000. Relationship between trunk cross‐sectional area, harvest index, total tree dry weight and yield components of ‘Starkspur Supreme Delicious’ apple trees. Journal of American Pomological Society 54: 22–27.

[nph17960-bib-0075] Takano J , Wada M , Ludewig U , Schaaf G , von Wirén N , Fujiwara T . 2006. The *Arabidopsis* major intrinsic protein NIP5;1 is essential for efficient boron uptake and plant development under boron limitation. Plant Cell 18: 1498–1509. 16679457 10.1105/tpc.106.041640 PMC1475503

[nph17960-bib-0076] Tardieu F . 2012. Any trait or trait‐related allele can confer drought tolerance: just design the right drought scenario. Journal of Experimental Botany 63: 25–31. 21963615 10.1093/jxb/err269

[nph17960-bib-0077] Tisné S , Schmalenbach I , Reymond M , Dauzat M , Pervent M , Vile D , Granier C . 2010. Keep on growing under drought: genetic and developmental bases of the response of rosette area using a recombinant inbred line population. Plant, Cell & Environment 33: 1875–1887. 10.1111/j.1365-3040.2010.02191.x 20545881

[nph17960-bib-0078] Turner NC . 2019. Imposing and maintaining soil water deficits in drought studies in pots. Plant and Soil 439: 45–55.

[nph17960-bib-0079] Urrestarazu J , Denancé C , Ravon E , Guyader A , Guisnel R , Feugey L , Poncet C , Lateur M , Houben P , Ordidge M *et al*. 2016. Analysis of the genetic diversity and structure across a wide range of germplasm reveals prominent gene flow in apple at the European level. BMC Plant Biology 16: 130. 27277533 10.1186/s12870-016-0818-0 PMC4898379

[nph17960-bib-0080] VanRaden PM . 2008. Efficient methods to compute genomic predictions. Journal of Dairy Science 91: 4414–4423. 18946147 10.3168/jds.2007-0980

[nph17960-bib-0081] Vargas WA , Pontis HG , Salerno GL . 2008. New insights on sucrose metabolism: evidence for an active A/N‐Inv in chloroplasts uncovers a novel component of the intracellular carbon trafficking. Planta 227: 795–807. 18034262 10.1007/s00425-007-0657-1

[nph17960-bib-0082] Virlet N , Costes E , Martinez S , Kelner J‐J , Regnard J‐L . 2015. Multispectral airborne imagery in the field reveals genetic determinisms of morphological and transpiration traits of an apple tree hybrid population in response to water deficit. Journal of Experimental Botany 66: 5453–5465. 26208644 10.1093/jxb/erv355 PMC4585425

[nph17960-bib-0083] Ward JM , Mäser P , Schroeder JI . 2009. Plant ion channels: gene families, physiology, and functional genomics analyses. Annual Review of Physiology 71: 59–82. 10.1146/annurev.physiol.010908.163204 PMC4790454 18842100

[nph17960-bib-0084] Willaume M , Lauri P‐É , Sinoquet H . 2004. Light interception in apple trees influenced by canopy architecture manipulation. Trees 18: 705–713.

[nph17960-bib-0085] Zhang D , Zhang Z , Yang K . 2006. QTL analysis of growth and wood chemical content traits in an interspecific backcross family of white poplar (*Populus tomentosa × P. bolleana*) × *P. tomentosa* . Canadian Journal of Forest Research 36: 2015–2023.

[nph17960-bib-0086] Zhou X , Stephens M . 2012. Genome‐wide efficient mixed‐model analysis for association studies. Nature Genetics 44: 821–824. 22706312 10.1038/ng.2310 PMC3386377

