# Supplementary material for: Tree architecture, light interception and water‐use related traits are controlled by different genomic regions in an apple tree core collection
Source: New Phytol. 2022 Feb 8;234(1):209–26. doi: 10.1111/nph.17960 (PMC9305758; doi:10.1111/nph.17960)
Supplement: Supplementary file 1 — Fig. S1 Evolution of soil water potential during the experiment. Fig. S2 Tree architecture and light‐interception related traits retrieved from T‐LiDAR measurements. Fig. S3 Vegetation indices (NDVI, GNDVI and MCARI2) are stable and correlated across dates and watering scenarios. Fig. S4 Canopy surface temperature (TSTA) under the well‐watered scenario across dates. Fig. S5 Correlations between the proportion of maximal canopy temperature increase observed at the light and moderate intensities of soil dryness, and the maximal canopy temperature increase. Fig. S6 Weak correlations between tree canopy temperature and traits related to vegetative architecture and light interception. Fig. S7 Two regions associated with traits related to light interception capacity on chromosomes 7 and 15. Fig. S8 Three regions associated with traits related to tree architecture on chromosomes 2, 4, and 6. Fig. S9 Boxplots of allelic effects for a large region associated with several architecture‐related traits on chromosome 13. Fig. S10 Heatmap of linkage disequilibrium in a large region associated with several architecture‐related traits on chromosome 13. Fig. S11 A large genomic region controls architecture‐related traits on chromosome 13. Fig. S12 Haplotype analysis of a genomic region controlling architecture‐related traits on chromosome 13. Fig. S13 Different regions controlling respectively the canopy temperature in well‐watered conditions, or its response to water deficit. Fig. S14 Heatmap of allelic effects for the 16 highly reliable associations and the 241 cultivars of the apple tree core‐collection. Methods S1 Haplotype analysis of a genomic region controlling architecture‐related traits on chromosome 13. Table S1 Summary of the meteorological variables during the drones’ flights at the four dates of measurements in July 2017. Table S2 Leaf and stem water potentials measured on 45 trees among the apple tree core‐collection. [file NPH-234-209-s001.pdf]

New Phytologist

New Phytologist Supporting Information

Article title: Tree architecture, light interception and water use related traits are controlled by different genomic regions in an apple tree core collection

Aude Coupel-Ledru, Benoît Pallas, Magalie Delalande, Vincent Segura, Baptiste Guitton, Hélène Muranty, Charles-Eric Durel, Jean-Luc Regnard, Evelyne Costes

Article acceptance date: 19 December 2021

The following Supporting Information is available for this article:

Fig. S1. Evolution of soil water potential during the experiment
Fig. S2. Tree architecture and light-interception related traits retrieved from T-LiDAR measurements.
Fig. S3. Vegetation indices (*NDVI*, *GNDVI* and *MCARI2*) are stable and correlated across dates and watering scenarios.
Fig. S4. Canopy surface temperature (*TSTA*) under the well-watered scenario across dates.
Fig. S5. Correlations between the proportion of maximal canopy temperature increase observed at the light and moderate intensities of soil dryness, and the maximal canopy temperature increase.
Fig. S6. Weak correlations between tree canopy temperature and traits related to vegetative architecture and light interception.
Fig. S7. Two regions associated with traits related to light interception capacity on chromosomes 7 and 15.
Fig. S8. Three regions associated with traits related to tree architecture on chromosomes 2, 4, and 6.
Fig. S9. Boxplots of allelic effects for a large region associated with several architecture-related traits on chromosome 13.
Fig. S10. Heatmap of linkage disequilibrium in a large region associated with several architecture-related traits on chromosome 13.
Fig. S11. A large genomic region controls architecture-related traits on chromosome 13.
Methods S1. Haplotype analysis of a genomic region controlling architecture-related traits on chromosome 13.
Fig. S12. Haplotype analysis of a genomic region controlling architecture-related traits on chromosome 13.
Fig. S13. The two SNPs significant for *resp.TSTA* had no effect on *TSTA.WW*.
Fig. S14. Heatmap of allelic effects for the 16 highly reliable associations and the 241 cultivars of the apple tree core-collection.
Table S1. Summary of the meteorological variables during the drones' flights at the four dates of measurements in July 2017.
Table S2. Leaf and stem water potentials measured on 45 trees among the apple tree core-collection.
Table S3. Complete set of associations detected for the traits related to vigour, light interception and canopy temperature response to water deficit computed from T-LiDAR, multispectral and thermal imaging on a collection of 241 apple tree varieties. *[This large table is provided as a separate Excel file]*.
Table S4. Summary of the GWAS results per trait.
Table S5. List of genes underlying the most highly reliable SNPs. *[This large table is provided as a separate Excel file]*.
Table S6. Co-localizations between associated SNPs on the apple core collection and SSR associated on bi-parental populations for similar or related traits.
Table S7. List of allelic effects for the 16 highly reliable associations and the 241 cultivars of the apple tree core-collection. *[This large table is provided as a separate Excel file]*.

Fig. S1. Evolution of soil water potential during the experiment. Soil water potential (Ψ_{soil}) was measured at 60 cm depth by Watermark® probes for a subset of 13 trees (7 well-watered, WW, and 6 water deficit, WD) randomly located in the field and monitored during summer 2017 in Montpellier. All trees were well-irrigated (2h everyday) until July 7th (black line). The irrigation was then limited to 2h twice a week for the WD trees. Mean ± SD calculated over the probes of each scenario, for each day. The dates when the airborne flights were carried out are indicated with black arrows.

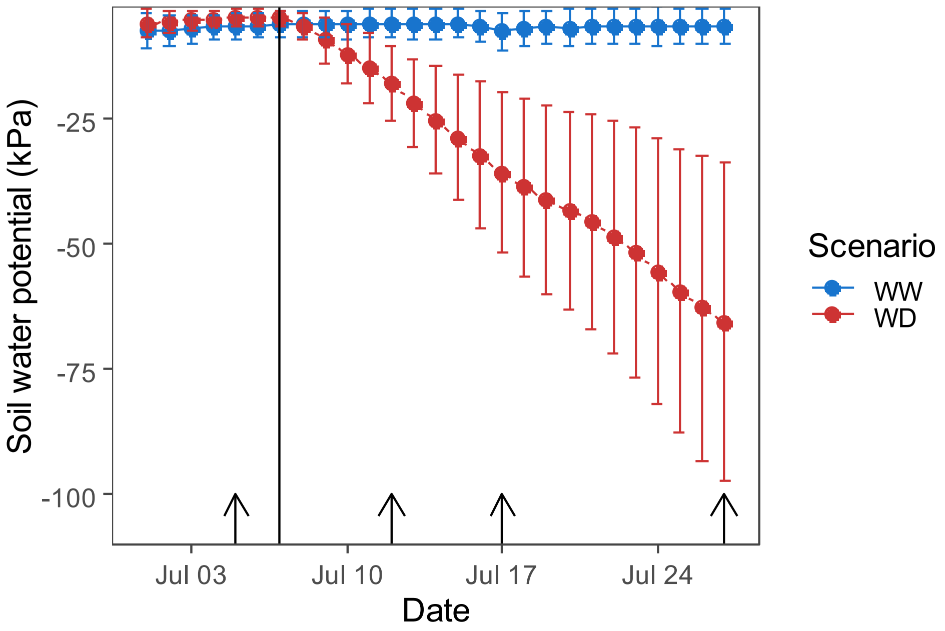

Fig. S2. Tree architecture and light-interception related traits retrieved from T-LiDAR measurements. (a) Examples of image outputs obtained for one tree from winter (left) and summer (right) T-LiDAR measurements. (b,c) Correlation coefficients (r) between variables and the first or second dimensions of the PCA performed on traits retrieved from T-LiDAR measurements (PCA projections are presented in Figure 1). The analysis was carried out on individual tree values. *n*=964 trees.

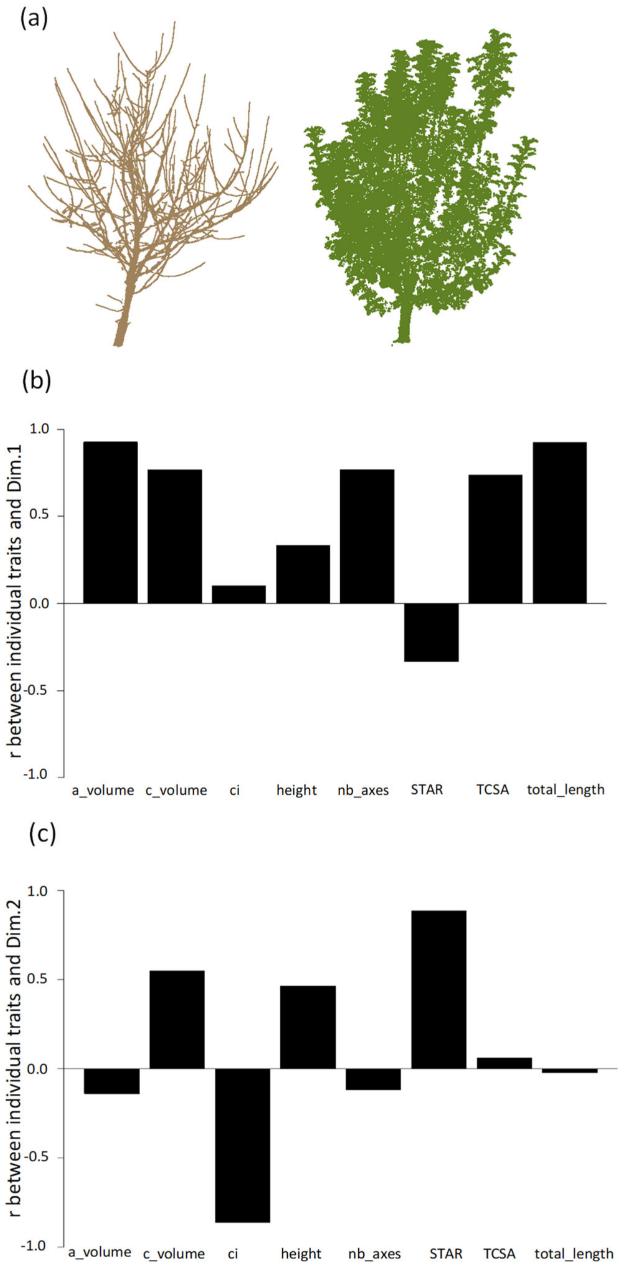

Fig. S3. Vegetation indices (*NDVI*, *GNDVI* and *MCARI2*) are stable and correlated across dates and watering scenarios. (a, b, c) Distribution and correlation between BLUPs of *NDVI* (respectively *MCARI2* and *GNDVI*) across 4 dates of measurements during the month of July and BLUPs computed considering all the dates together (all.dates). WW: well-watered, WD: water deficit, all: both scenarios together. BLUPs for *n*=241 cultivars. Pearson's correlation coefficients are indicated. All correlations are highly significant (*pval*<10^{-4}).

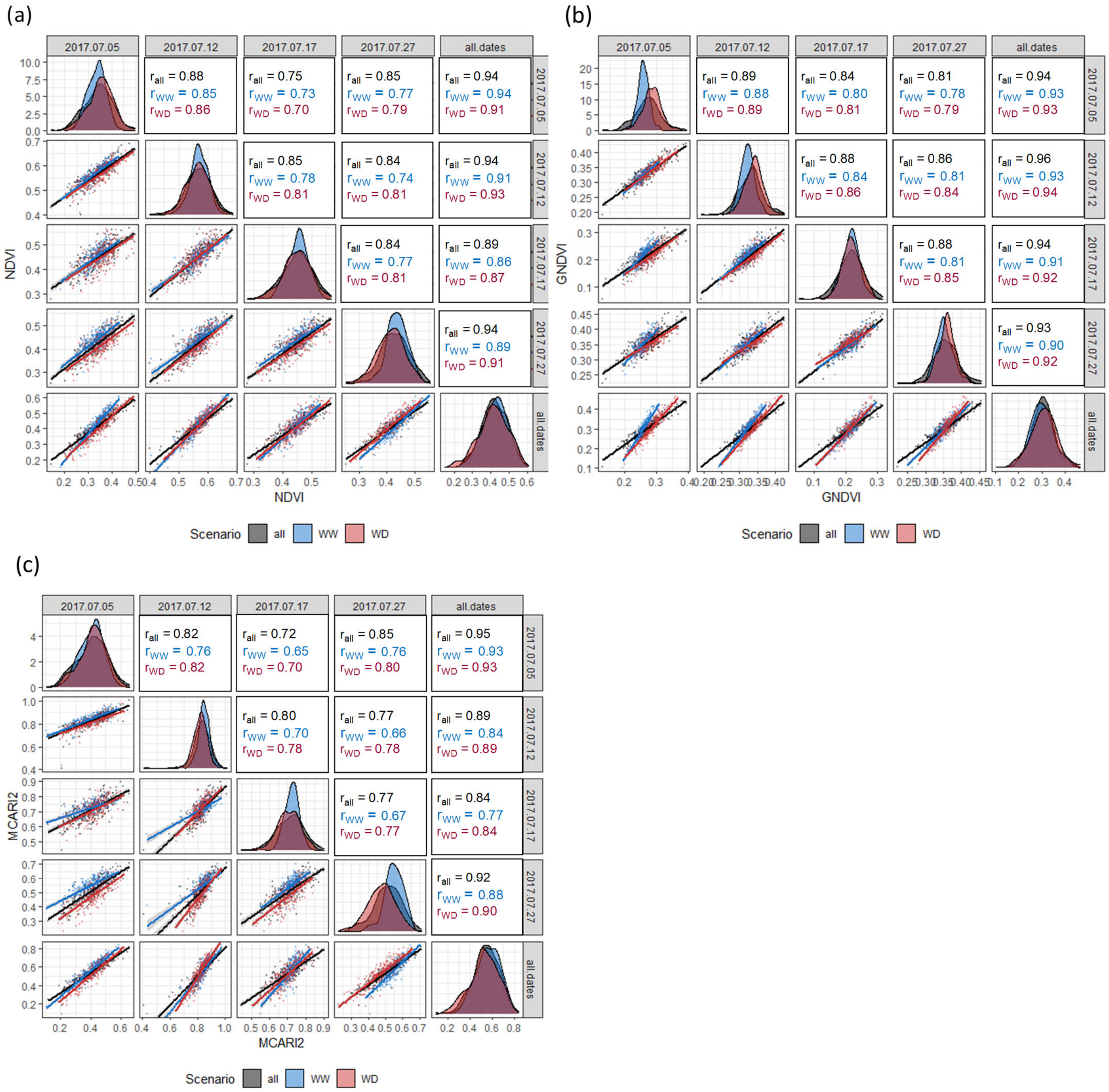

Fig. S4. Canopy surface temperature (*TSTA*) under the well-watered (WW) scenario across dates. *TSTA* values represent the difference between canopy and air temperature and were computed from airborne thermal imaging. Distributions and correlations for the BLUPs across the four dates of measurements, and for the BLUPs calculated over all dates (all.dates). *n*=241 cultivars. Boxplots present the median (centre horizontal line) and interquartile range (blue box) of each group. The upper and lower whiskers represent data within 1.5 * the interquartile range, and values beyond these upper and lower bounds are considered outliers, marked with dots. Pearson's correlation coefficients are displayed together with their significance (*** pval<10^{-3}, ** pval<10^{-2}, * pval<0.05).

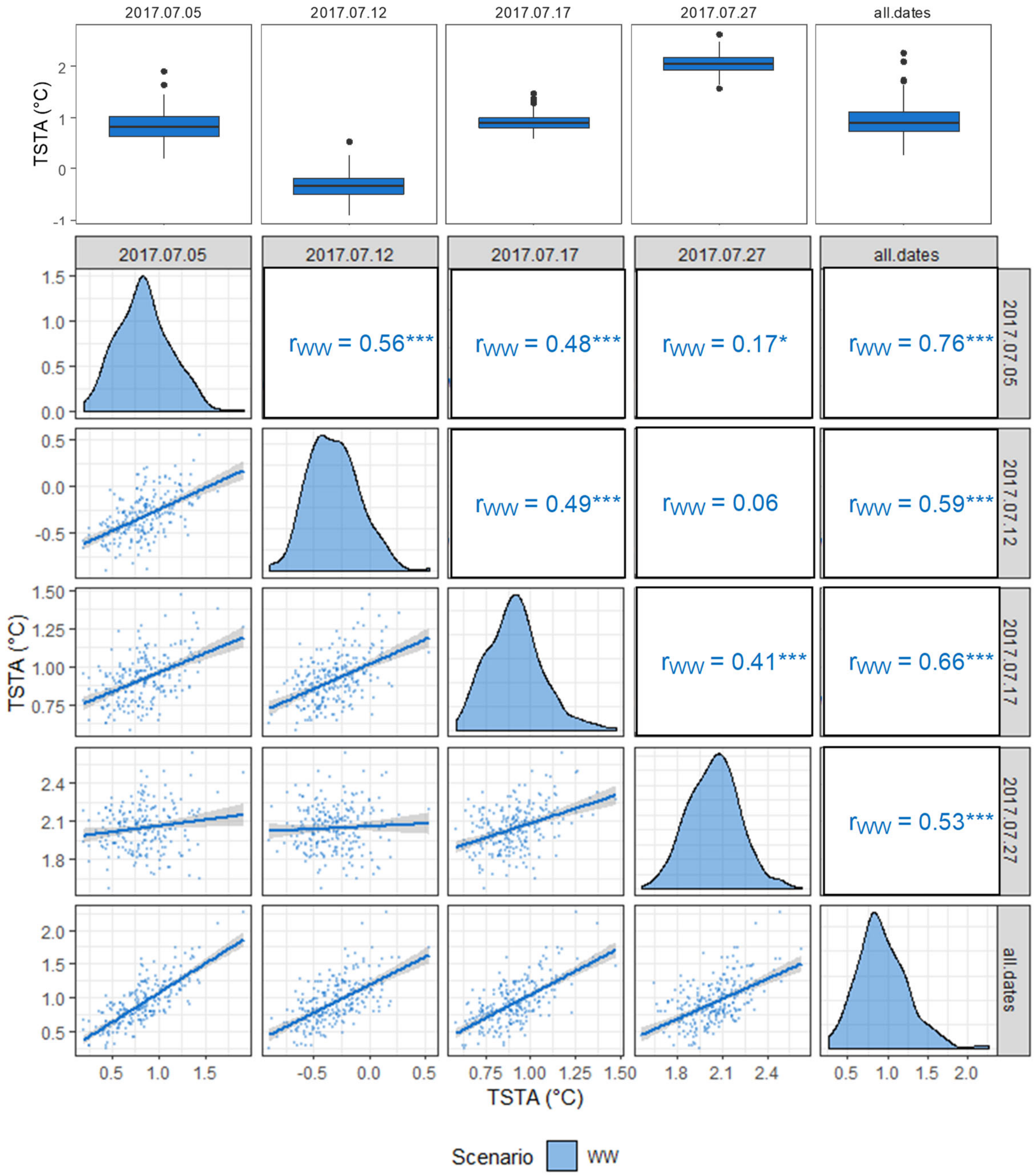

Fig. S5. Correlations between the proportion of maximal canopy temperature response observed at the light and moderate intensities of soil dryness, and the maximal canopy temperature response. (a) Correlations between the proportion of maximal canopy temperature response observed at the light intensity of soil dryness (*contrib_12.07*) and the response of canopy temperature observed at the severe intensity of soil dryness (*resp.TSTA_27.07*). (b) Correlations between the proportion of maximal canopy temperature response observed at the moderate intensity of soil dryness (*contrib_17.07*) and the maximal canopy temperature response observed at the severe intensity of soil dryness (*resp.TSTA_27.07*). *n*=241 cultivars. 'ns', non-significant.

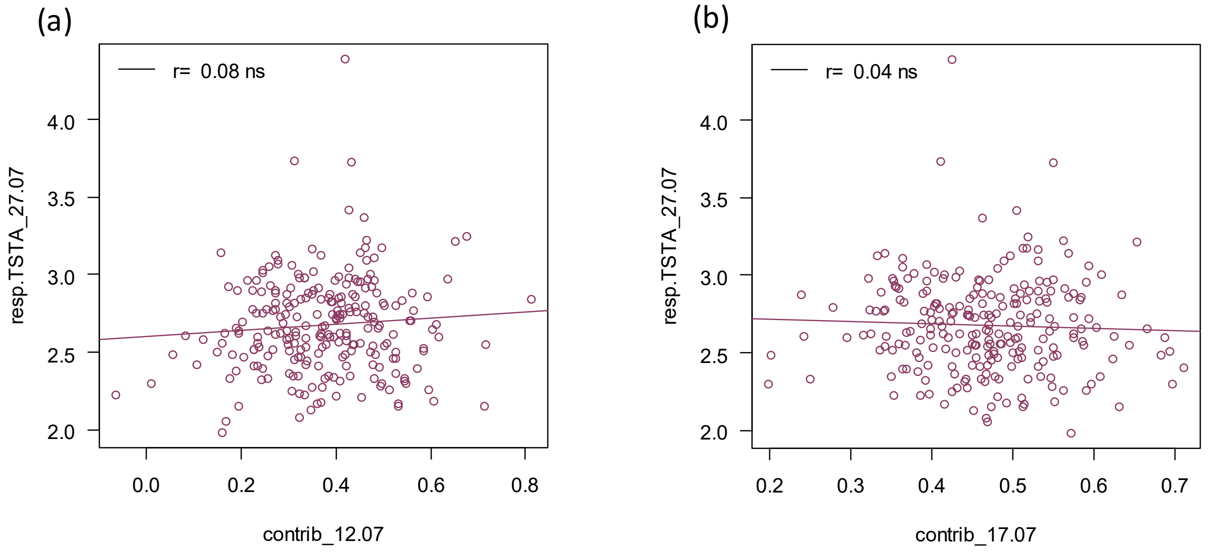

Fig. S6. Weak correlations between tree canopy temperature and traits related to vegetative architecture and light interception. (a, b, e, f) Relationship between BLUPs of canopy temperature in well-watered conditions (*TSTA.WW)* and individual traits representing vegetative development (*a_volume*, (a); *total_length*, (e)) or light interception (*ci*, (b); *STAR*, (f)). (c, d, g, h) Relationship between BLUPs of the response of canopy temperature to severe water deficit (*resp.TSTA_27.07)* and individual traits representing vegetative development (*a_volume*, (c); *total_length*, (g)) or light interception (*ci*, (d); *STAR*, (h)). *n*=241 cultivars. Pearson's correlation coefficients are displayed together with their significance (*** *pval*$<10^{-3}$, * pval<0.05). *a_volume*, alpha hull volume; *total_length*, total cumulative axis length; *STAR*, silhouette to leaf area ratio; c*i*, convexity index; *Dim.1* and *Dim.2*, respectively first and second principal components of the PCA analysis on light interception, plant architecture and shoot traits.

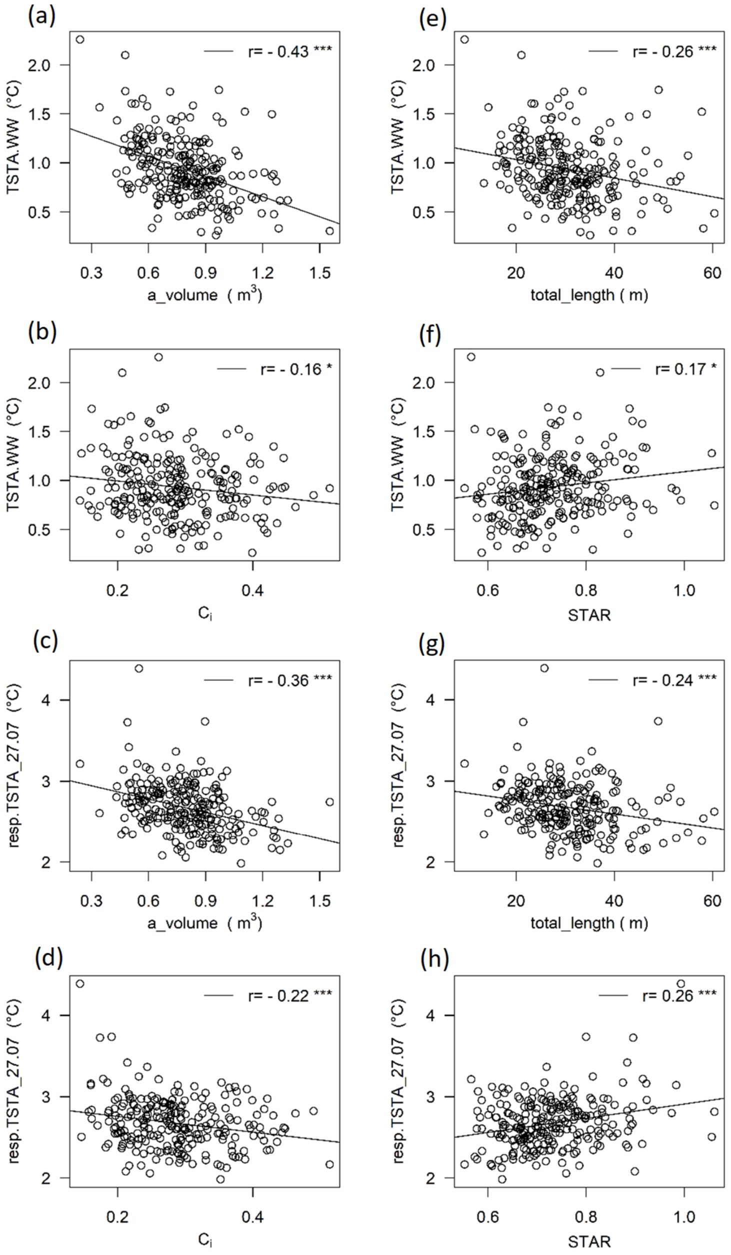

Fig. S7. Two regions associated with traits related to light interception capacity on chromosomes 7 and 15. (a) SNP AX-115510439, found significant for *Dim.2*, also displayed substantial effects on *STAR* and *ci*. (b) SNP AX-115209148, found significant for *ci*, also displayed substantial effects on *STAR* and *Dim.2*. Boxplots present the median (centre horizontal line) and interquartile range (grey box) of each group. The upper and lower whiskers represent data within 1.5 * the interquartile range, and values beyond these upper and lower bounds are considered outliers, marked with dots. The $-\log_{10}(pval)$ from SNP-by-SNP GWAS with GEMMA is indicated in each boxplot, and a star (*) is added if significant ($-\log_{10}(pval)$ >5.63). The mention 'MLMM: *' is added if the SNP is retained as significant in the multi-SNP GWAS with MLMM. *STAR*, silhouette to leaf area ratio; *ci*, convexity index; *Dim.2*, second principal component of the PCA analysis on light interception, plant architecture and shoot traits, mostly associated with light interception.

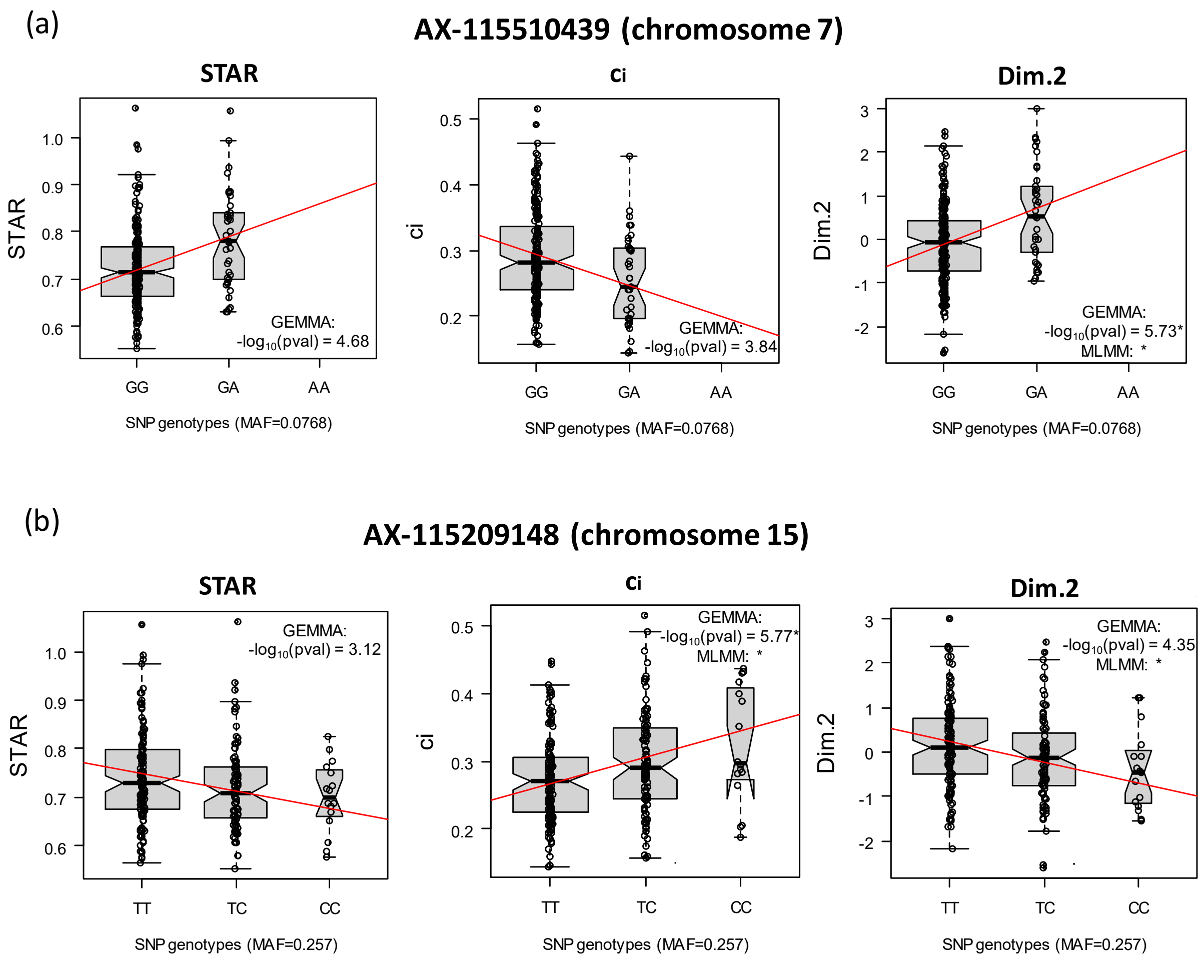

Fig. S8. Three regions associated with traits related to tree architecture on chromosomes 2, 4, and 6. (a) On chromosome 2, SNP AX-115511400 significant for *TCSA*, also displayed substantial effects on *Dim.1*. (b) On chromosome 4, SNP AX-115374200 significant for *Dim.1*, also displayed substantial effects on *total_length* and SNP AX-10521494 significant for *total_length* also displayed substantial effects on *Dim.1*. Both SNPs were in high LD (R^2=0.54). (c) On chromosome 6, SNP AX-115471271 significant for *MCARI2*, also displayed substantial effects on *NDVI* and SNP AX-115491300 significant for *NDVI* also displayed substantial effects on *MCARI2*. Both SNPs were in high LD (R^2=0.97). Boxplots present the median (centre horizontal line) and interquartile range (grey box) of each group. The upper and lower whiskers represent data within 1.5 * the interquartile range, and values beyond these upper and lower bounds are considered outliers, marked with dots. The -$\log_{10}$(*pval*) from SNP-by-SNP GWAS with GEMMA is indicated in each boxplot, and a star (*) is added if significant (-$\log_{10}$(*pval*)>5.63). The mention 'MLMM: *' is added if the SNP is retained as significant in the multi-SNP GWAS with MLMM. *total_length*, total cumulative axis length; *TCSA*, trunk cross sectional area; *Dim.1*, first principal component of the PCA analysis on light interception, plant architecture and shoot traits, mostly associated with vegetative development.

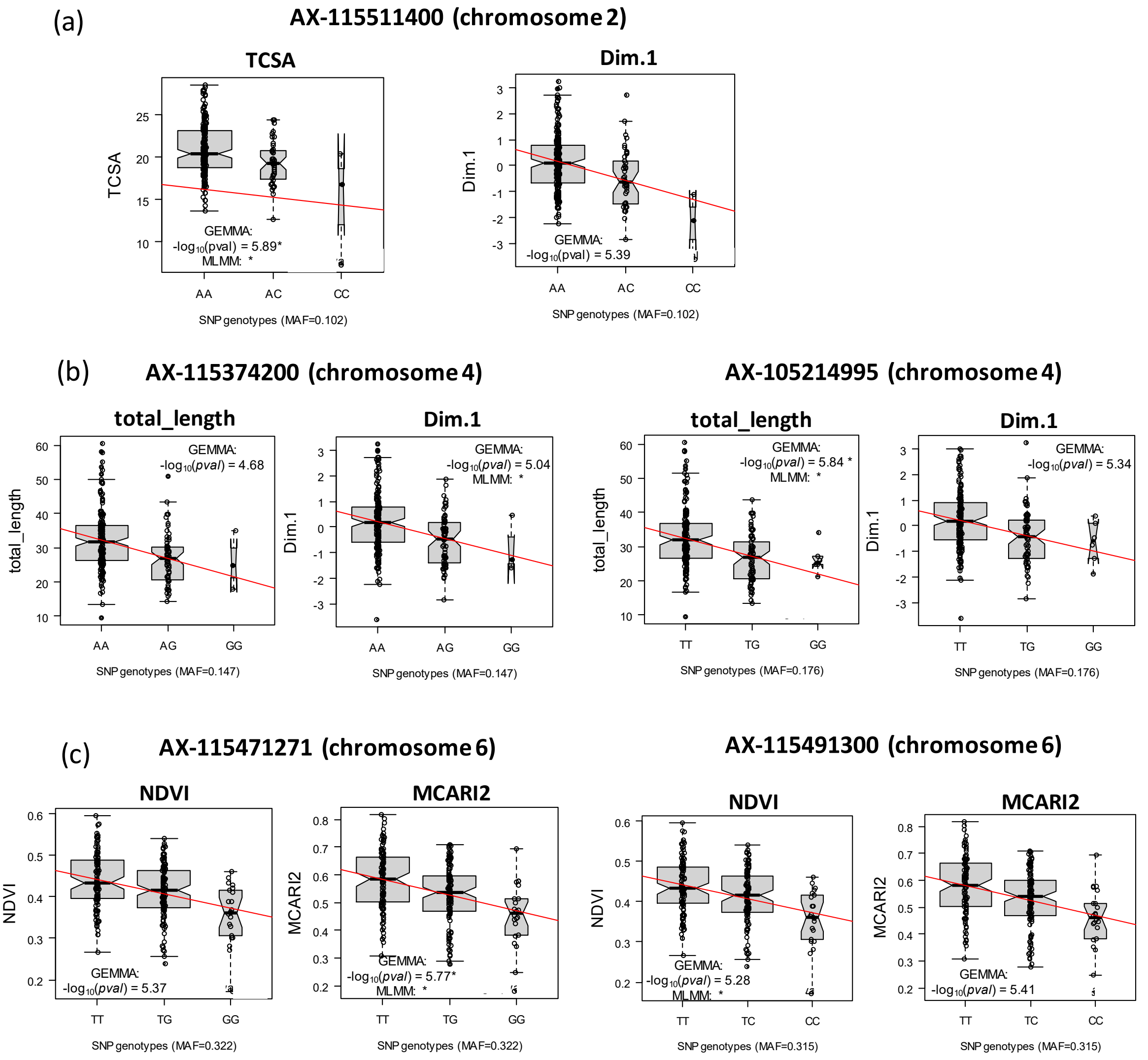

Fig. S9. Boxplots of allelic effects for a large region associated with several tree architecture-related traits on chromosome 13. (a-n) Boxplots for 14 unique SNPs detected within a 1 Mb region of Chromosome 13 for *a_volume* (2 SNPs), *Dim.1* (2 SNPs), *GNDVI* (10 SNPs), *MCARI2* (5 SNPs) and *NDVI* (13 SNPs). Boxplots present the median (centre horizontal line) and interquartile range (grey box) of each group. The upper and lower whiskers represent data within 1.5 * the interquartile range, and values beyond these upper and lower bounds are considered outliers, marked with dots. The $-\log_{10}(pval)$ from SNP-by-SNP GWAS with GEMMA is indicated in each boxplot, and a star (*) is added if significant ($-\log_{10}(pval)>5.63$). The mention 'MLMM: *' is added if the SNP is retained as significant in multi-SNP GWAS with MLMM. *a_volume*, alpha hull volume; *Dim.1*, first principal component of the PCA analysis on light interception, plant architecture and shoot traits, mostly associated with vegetative development.

1/2

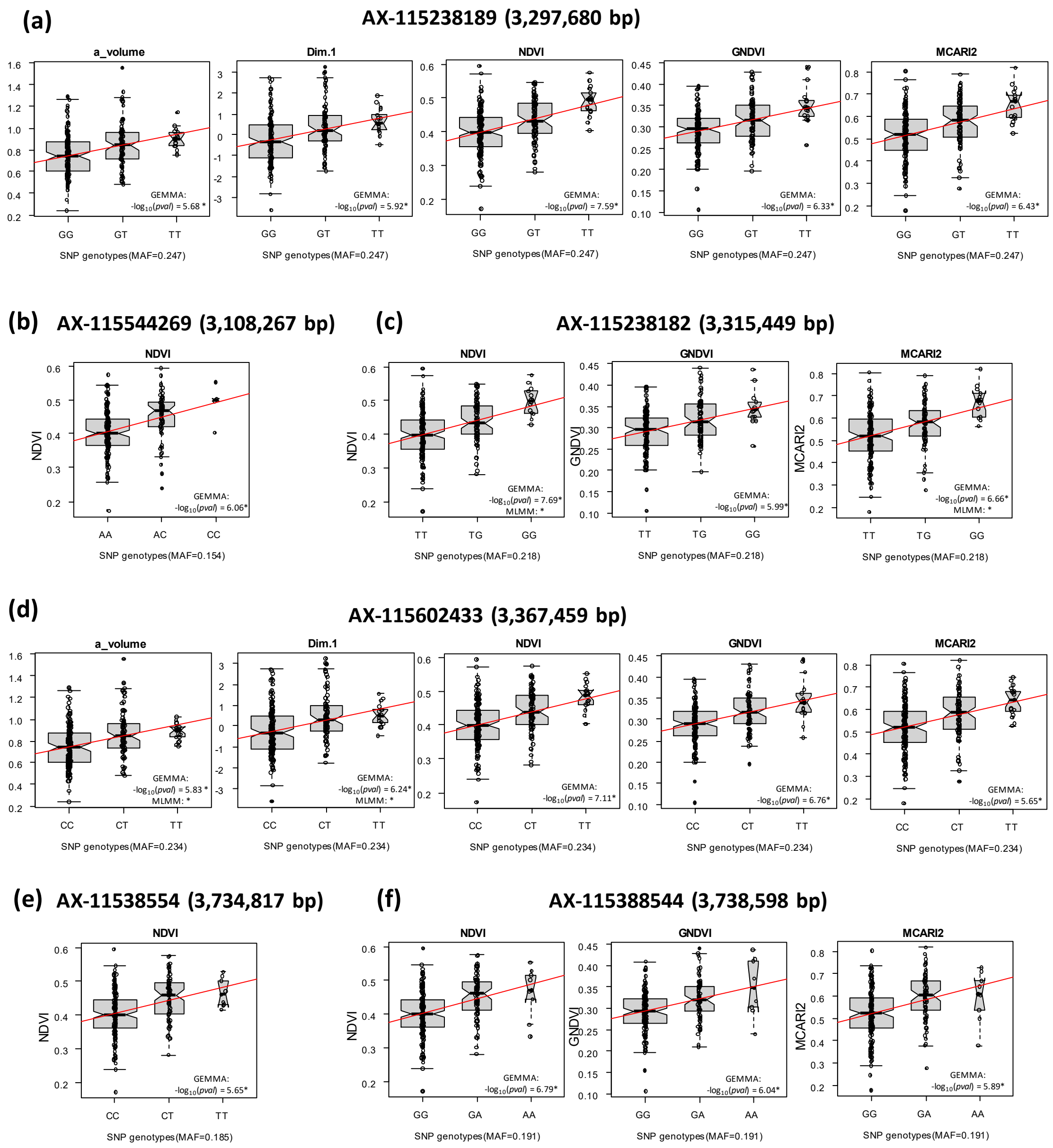

Fig. S9.

2/2

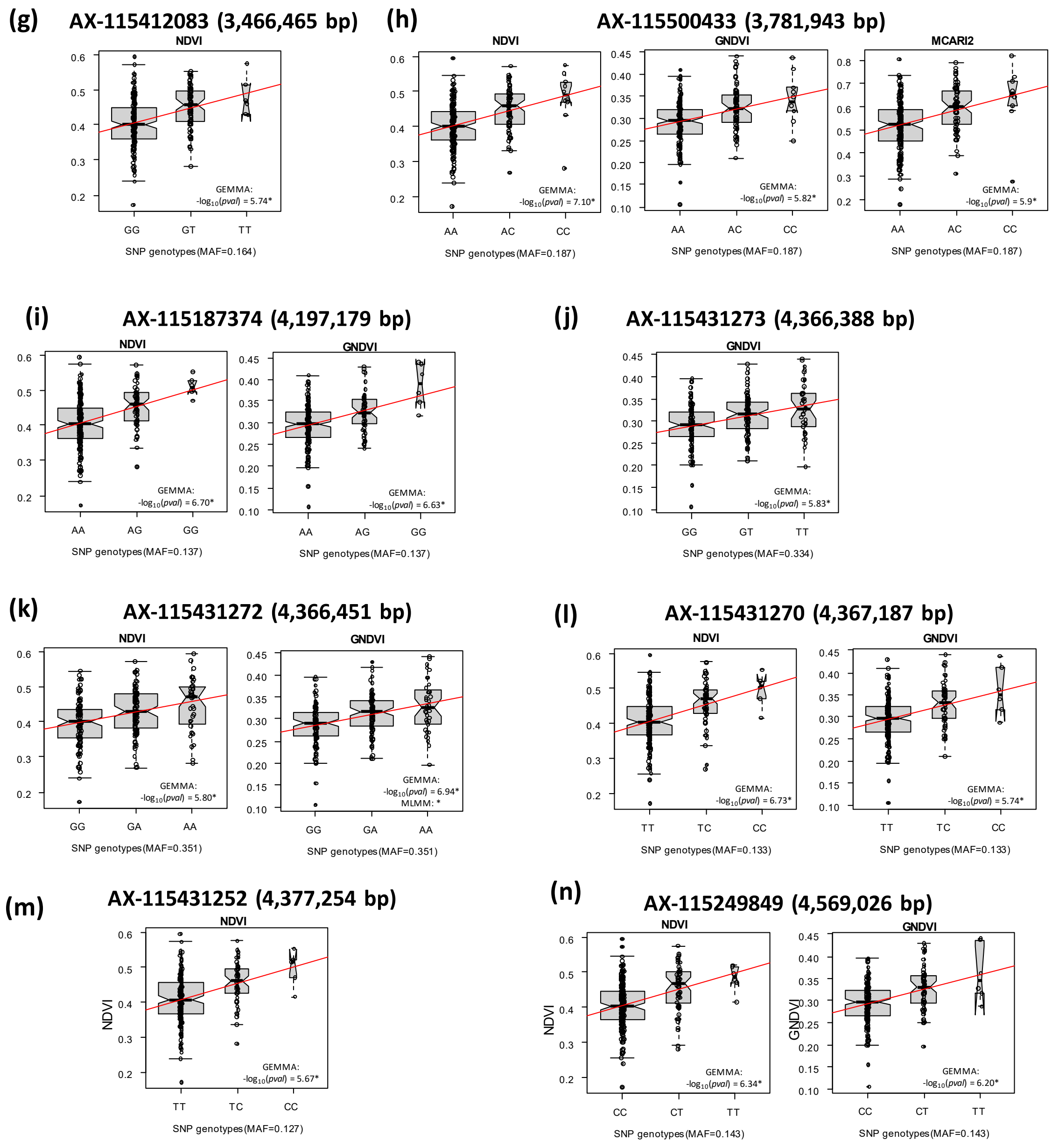

(g)
AX-115412083 (3,466,465 bp)
NDVI
GEMMA: -log10(pval) = 5.74*
GG GT TT
SNP genotypes(MAF=0.164)
(h)
AX-115500433 (3,781,943 bp)
NDVI
GEMMA: -log10(pval) = 7.10*
AA AC CC
SNP genotypes(MAF=0.187)
GNDVI
GEMMA: -log10(pval) = 5.82*
AA AC CC
SNP genotypes(MAF=0.187)
MCARI2
GEMMA: -log10(pval) = 5.9*
AA AC CC
SNP genotypes(MAF=0.187)
(i)
AX-115187374 (4,197,179 bp)
NDVI
GEMMA: -log10(pval) = 6.70*
AA AG GG
SNP genotypes(MAF=0.137)
GNDVI
GEMMA: -log10(pval) = 6.63*
AA AG GG
SNP genotypes(MAF=0.137)
(j)
AX-115431273 (4,366,388 bp)
GNDVI
GEMMA: -log10(pval) = 5.83*
GG GT TT
SNP genotypes(MAF=0.334)
(k)
AX-115431272 (4,366,451 bp)
NDVI
GEMMA: -log10(pval) = 5.80*
GG GA AA
SNP genotypes(MAF=0.351)
GNDVI
GEMMA: -log10(pval) = 6.94*
MLMM: *
GG GA AA
SNP genotypes(MAF=0.351)
(l)
AX-115431270 (4,367,187 bp)
NDVI
GEMMA: -log10(pval) = 6.73*
TT TC CC
SNP genotypes(MAF=0.133)
GNDVI
GEMMA: -log10(pval) = 5.74*
TT TC CC
SNP genotypes(MAF=0.133)
(m)
AX-115431252 (4,377,254 bp)
NDVI
GEMMA: -log10(pval) = 5.67*
TT TC CC
SNP genotypes(MAF=0.127)
(n)
AX-115249849 (4,569,026 bp)
NDVI
GEMMA: -log10(pval) = 6.34*
CC CT TT
SNP genotypes(MAF=0.143)
GNDVI
GEMMA: -log10(pval) = 6.20*
CC CT TT
SNP genotypes(MAF=0.143)

Fig. S10. Heatmap of linkage disequilibrium in a large region associated with several architecture-related traits on chromosome 13. The 14 SNPs significant with GEMMA for several traits are mentioned in blue letters.

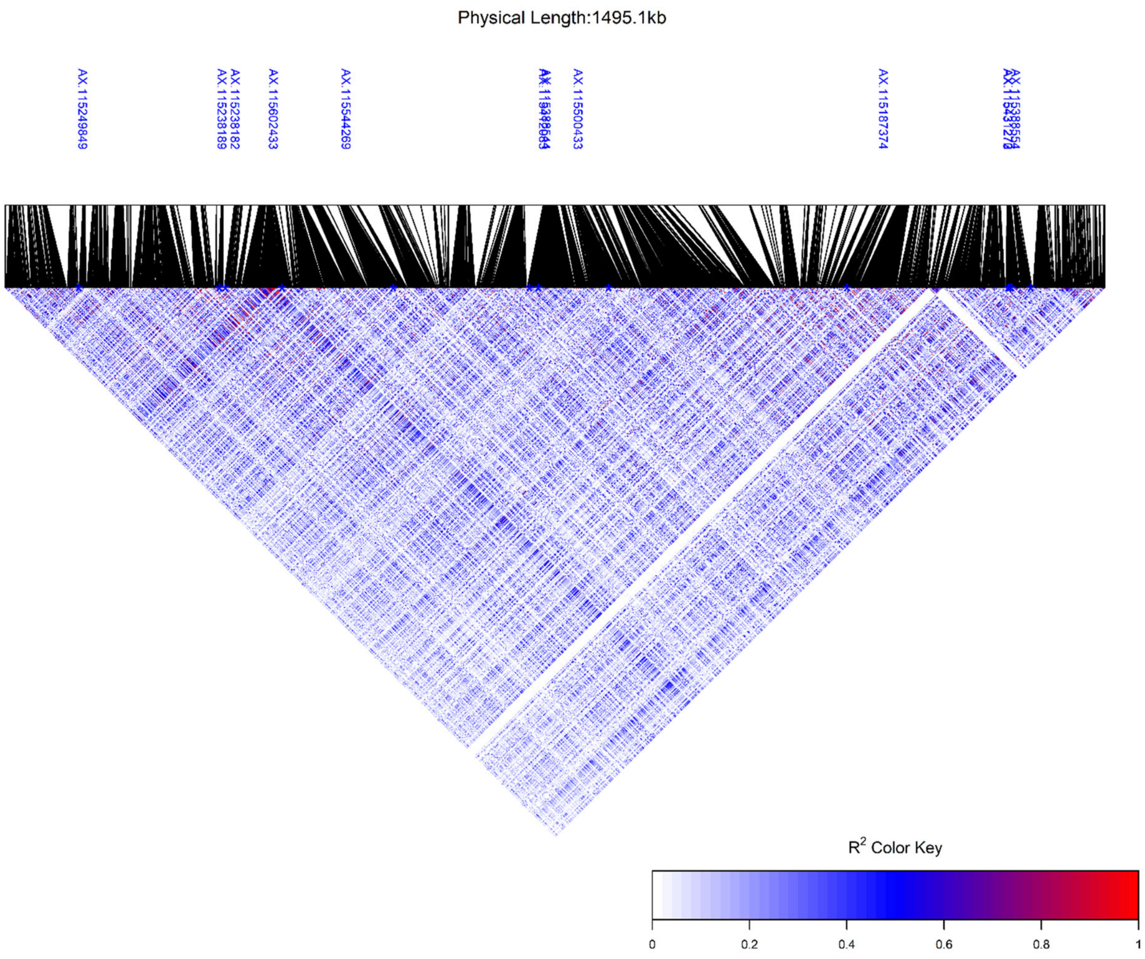

Fig. S11. A large genomic region controls tree architecture-related traits on chromosome 13. Boxplots of BLUPs of *Dim.1*, *GNDVI* and *MCARI2* per group determined from the clustering analysis carried out on allelic values for the 14 SNPs detected on chromosome 13 and 241 cultivars. Group 1, *n*=183 cultivars; group 2, *n*= 53 cultivars; group 3, *n*=5 cultivars. Boxplots present the median (centre horizontal line) and interquartile range (grey box) of each group. The upper and lower whiskers represent data within 1.5 * the interquartile range, and values beyond these upper and lower bounds are considered outliers, marked with dots. The significance of the group effect is displayed on the bottom right corner (*, 0.05<*pval*<0.01; ***, *pval*<0.001).

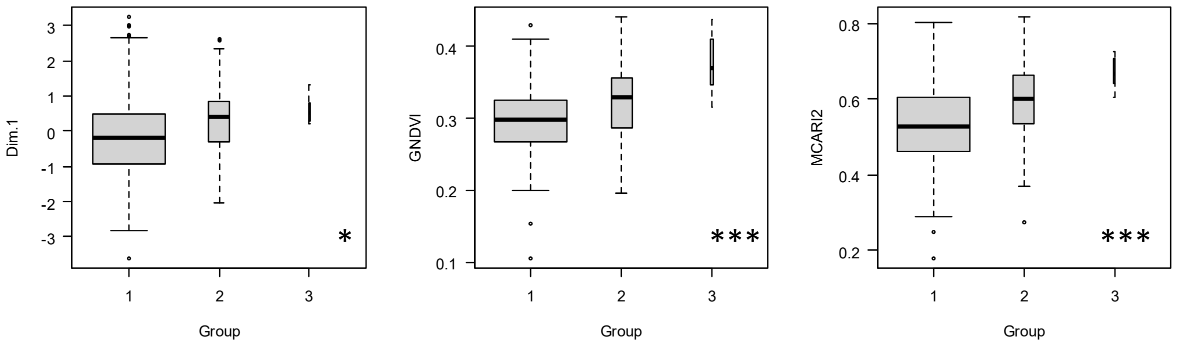

Methods S1. Haplotype analysis of a genomic region controlling architecture-related traits on chromosome 13. A large genomic region (3,210,000 - 4,469,999 bp) was found on chromosome 13 associated with several traits related to tree architecture. In order to complement the exploration of this region, it was subdivided into 126 contiguous haploblocks of 10kb (with 1-31 SNPs per haploblock). The haploalleles were inferred for each cultivar at each haploblock using phased SNPs data from the imputation task of Cazenave *et al.* (https://biorxiv.org/cgi/content/short/2021.08.27.457920v1) on a large set of ~1,300 apple cultivars including the present 241 cultivars. The number of different haploalleles per haploblock was then determined and ranged from 2 to 56. For each trait (*a_volume, Dim.1, MCARI2, NDVI, GNDVI*), we proceeded to an ANOVA at the haplotype level for each haploblock followed by a post-hoc Tukey multiple comparison test.

Figure S12. Haplotype analysis of a large genomic region controlling architecture-related traits on chromosome 13. The analysis was performed on the region 3,210,000 - 4,469,999 bp (Methods S1). (a) Main results of the analysis for the 5 traits that were found associated in this region (Fig. 5). Traits are ordered vertically from those most representative of tree vigour (top) to those most representative of leaf chlorophyll content (bottom). Manhattan plots (left side) from an ANOVA performed on each of the 126 haploblocks are represented. For each trait, the two most significant haploblocks are coloured and labeled. Boxplots of the haploalleles effects are represented on the right. Data labelled with the same letter cannot be statistically distinguished at $p < 0.05$ (post-hoc Tukey multiple comparison test). Haploblocks CHR13_BL9 and CHR13_BL11 were the most significant for *a_volume*, *Dim.1*, *MCARI2* and *NDVI*. BL11 contains the SNP AX-115238182 retained by MLMM for these traits. Conversely, CHR13_BL112 and CHR13_BL106 were the most significant for *GNDVI*; BL112 is located ~40kb apart from the SNP AX-115431272 retained by MLMM for *GNDVI*. (b) Barplots of genotypes distribution within each haploallele for the 4 haploblocks of interest.

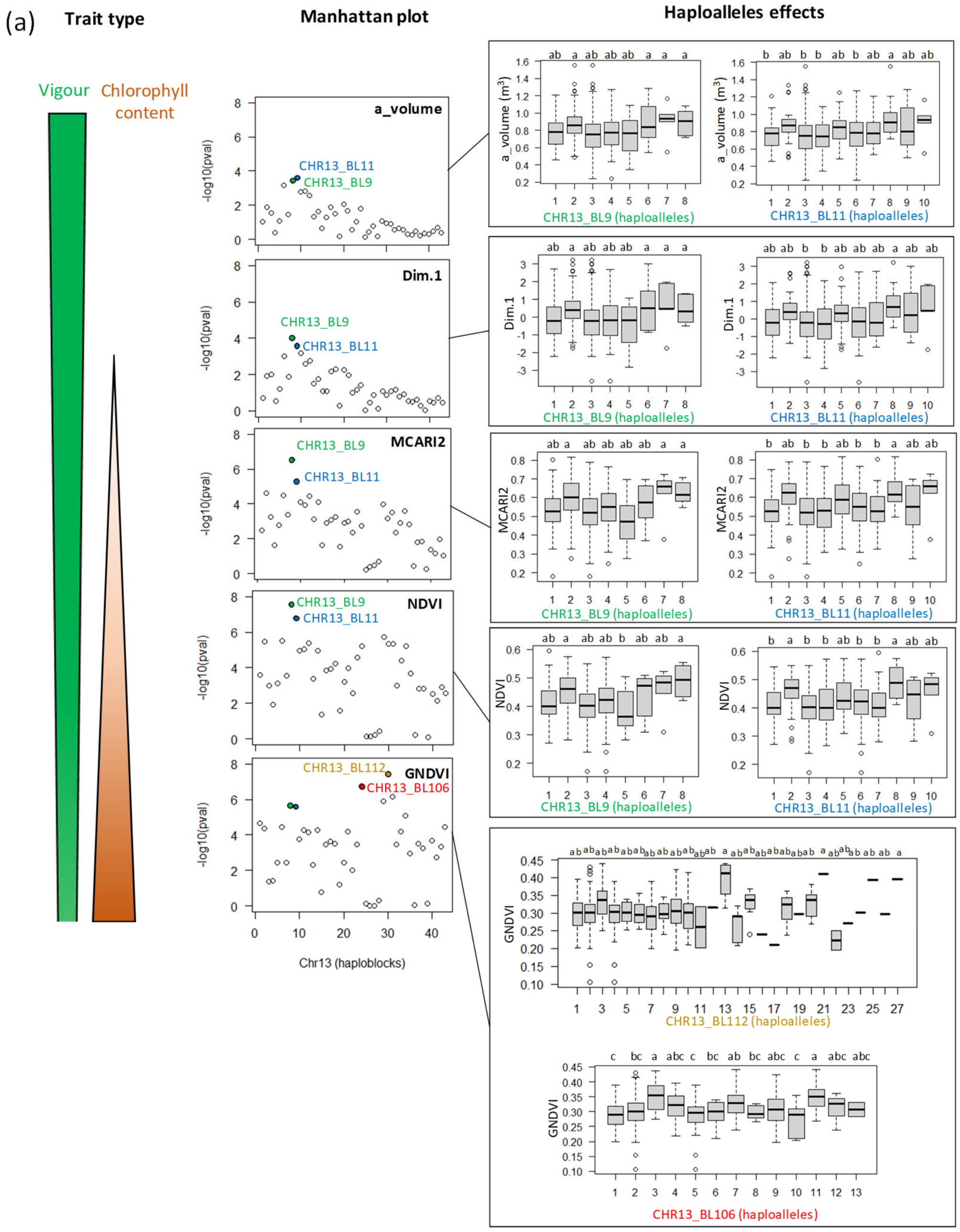

Figure S12. (2/2)

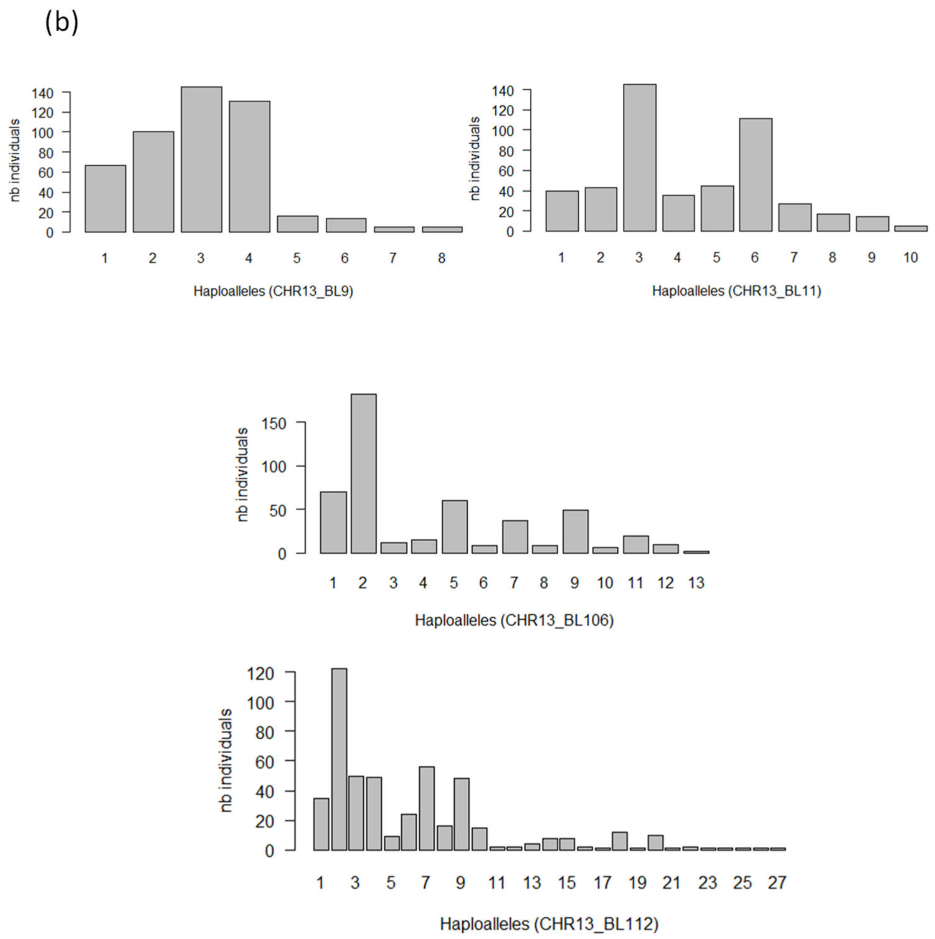

Fig. S13. The two SNPs significant for *resp.TSTA* had no effect on *TSTA.WW*. Boxplot of allelic effects. Boxplots present the median (centre horizontal line) and interquartile range (grey box) of each group. The upper and lower whiskers represent data within 1.5 * the interquartile range, and values beyond these upper and lower bounds are considered outliers, marked with dots. The -log$_{10}$(*pval*) from SNP-by-SNP GWAS with GEMMA is indicated in each boxplot, and a star (*) is added if significant. The mention 'MLMM: *' is added if the SNP is retained as significant with MLMM. 'NA', not available. *TSTA.WW* is in °C.

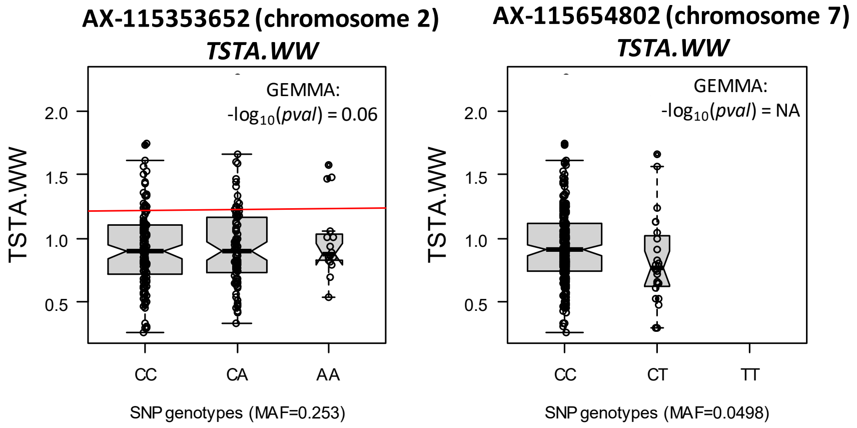

Fig. S14. Heatmap of allelic effects for the 16 highly reliable associations and the 241 cultivars of the apple tree core-collection. This figure allows a visual representation of data presented in Table S7.

The heatmap represents for each highly reliable SNP (listed in Table 2), the effect of the allele carried by each cultivar. 'Effect +', positive effect of the SNP on the trait; 'effect -', negative effect of the SNP on the trait; 'effect +/-', heterozygous cultivar. The 16 SNPs are ordered per type of trait for which they are highly reliable (vegetative architecture, vegetation indices, light interception, canopy surface temperature). A hierarchical clustering of cultivars was performed, and colored rectangle on the left represent the four first groups from the hierarchical clustering.

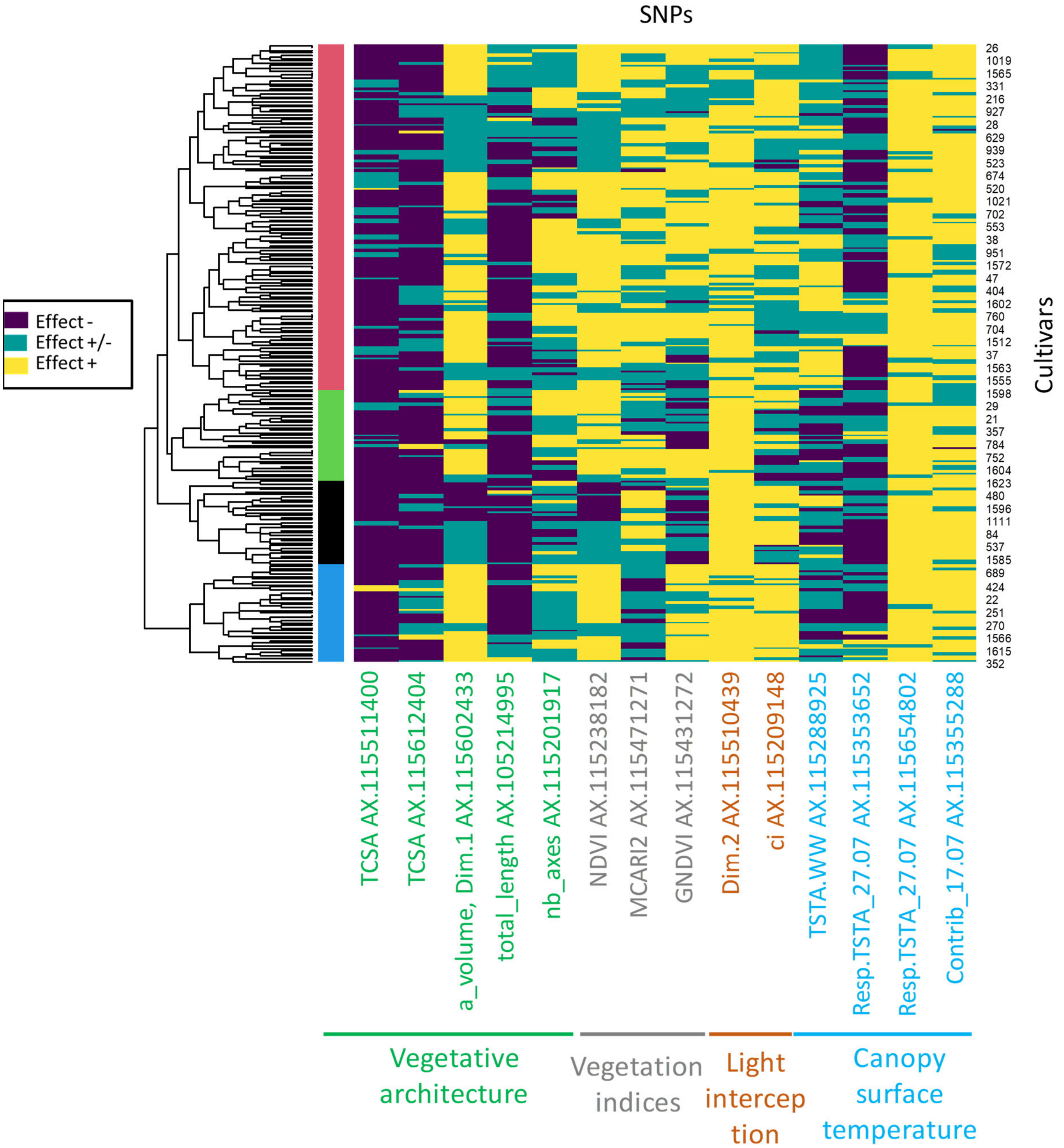

Table S1. Summary of the meteorological variables during the drones' flights at the four dates of measurements in July 2017. Flights were carried out on 5th, 12th, 17th and 27th July 2017. For each date, the flight was composed of three successive elevations of about 10 minutes each to cover the entire orchard (see Coupel-Ledru *et al.*, 2019 for details). Start and end time of each elevation (UTC) are reported together with mean, max and min air temperature (Air_Temp, °C), relative humidity (RH, %), vapour pressure deficit (VPD, kPa) and irradiance (Irradiance, W m^{-2}).

Date	Elevation	Time		Air_Temp			RH			VPD_mean			Irradiance		
		start	end	mean	max	min	mean	max	min	mean	max	min	mean	max	min
05/07/2017	1	10:18	10:27	22.39	22.59	22.14	73.9	74.82	72.98	0.71	0.74	0.68	916.8	922.8	907.7
	2	10:43	10:54	22.47	22.9	22.13	74.14	75.3	72.25	0.71	0.78	0.66	946.4	953.5	942.0
	3	11:09	11:20	22.72	23.02	22.45	72.68	74.53	71.31	0.76	0.81	0.69	965.5	968.8	961.5
12/07/2017	1	09:47	09:57	30.1	30.4	29.82	45.95	47.17	44.81	2.32	2.41	2.23	827.3	831.0	823.3
	2	10:12	10:24	31.86	32.43	31.59	35.27	36.06	34.72	3.07	3.19	2.99	874.4	878.8	867.5
	3	10:39	10:50	30.92	31.68	30.55	45.23	46.01	43.69	2.46	2.63	2.38	900.9	904.7	895.2
17/07/2017	1	09:46	09:54	27.6	27.84	27.28	35.1	35.97	34.21	2.41	2.47	2.35	838.8	842.5	834.7
	2	10:11	10:23	27.72	28.15	27.3	38.53	41.23	35.93	2.3	2.45	2.15	878.8	886.3	872.2
	3	10:37	10:49	27.45	27.74	26.97	45.71	47.57	44.47	2	2.08	1.87	910.6	915.0	903.8
27/07/2017	1	12:54	13:03	32.98	33.51	32.38	28.86	31.9	24.77	3.59	3.91	3.33	903.0	915.2	882.7
	2	13:18	13:30	30.16	30.63	29.71	38.4	39.3	37.46	2.65	2.76	2.55	868.2	875.5	852.2
	3	13:54	14:05	30.26	30.4	29.99	39.28	40.11	38.44	2.63	2.68	2.55	810.4	830.7	785.1

Table S2. Leaf and stem water potentials measured on 45 trees among the apple tree core-collection. Predawn leaf water potential ($\Psi_{leaf,\ predawn}$) and midday leaf ($\Psi_{leaf,\ midday}$) and stem ($\Psi_{stem,\ midday}$) water potentials were measured on 45 trees (22 well-watered, WW and 23 water deficit, WD) randomly distributed across the orchard at four dates during the month of July 2017 with two Schölander pressure chambers (Soil Moisture Equipment Corp., Santa Barbara, CA, USA). For each type of measurement, mean, standard deviation, minimum and maximum values are reported.

Date	Scenario	$\Psi_{leaf,\ predawn}$ (Mpa)				$\Psi_{leaf,\ midday}$ (Mpa)				$\Psi_{stem,\ midday}$ (Mpa)			
		Mean	SD	Min	Max	Mean	SD	Min	Max	Mean	SD	Min	Max
05/07/2017	WW	-0.071	0.018	-0.12	-0.035	-0.88	0.192	-1.32	-0.52	-0.542	0.079	-0.75	-0.42
05/07/2017	WD	-0.07	0.015	-0.1	-0.05	-0.923	0.224	-1.4	-0.44	-0.547	0.108	-0.95	-0.4
12/07/2017	WW	-0.091	0.015	-0.12	-0.06	-1.632	0.381	-2.53	-0.82	-1.073	0.31	-2.26	-0.68
12/07/2017	WD	-0.113	0.021	-0.15	-0.08	-1.871	0.334	-2.68	-1.27	-1.242	0.292	-2.02	-0.75
17/07/2017	WW	-0.073	0.014	-0.105	-0.05	-1.305	0.28	-2.22	-0.92	-0.85	0.175	-1.42	-0.66
17/07/2017	WD	-0.163	0.052	-0.3	-0.09	-1.652	0.279	-2.36	-1.17	-1.218	0.279	-2.02	-0.75
28/07/2017	WW	-0.191	0.04	-0.26	-0.05	-1.637	0.363	-2.4	-0.81	-1.099	0.325	-2	-0.55
28/07/2017	WD	-0.554	0.339	-1.52	-0.28	-2.118	0.431	-3.02	-1.21	-1.853	0.423	-2.85	-0.96

Table S3. Complete set of associations detected for the traits related to tree vegetative architecture, light interception and canopy temperature response to water deficit computed from T-LiDAR, multispectral and thermal imaging on a collection of 241 apple tree varieties. *[This large table is provided as a separate Excel file].* GWAS was performed on individual traits with a single-SNP method (GEMMA) and a multi-SNP method (MLMM). One row per SNP found significant with at least one method. For each association, the trait is indicated together with the SNP name, its location on a chromosome and its physical position (in bp). Physical positions are based on the apple genome GDDH13 v1.1. A SNP was declared significant with GEMMA ('signif GEMMA') if its $-\log_{10}(pval)$ ('$\log_{10}(pval)$ GEMMA') was above the Bonferroni threshold calculated from independent SNPs ($-\log_{10}(pval)>5.63$). MLMM was run with a maximum number of 7 steps for the stepwise regression, and a SNP was declared significant ('signif MLMM') if it was part of the selected model (the model with the greatest number of SNPs which all have a *p*-value smaller than the previously defined significance threshold (mBonf criterion). For each SNP, complementary columns indicate its minor allelic frequency ('MAF'), the minor and major alleles ('allele0' and 'allele1' respectively), and the allelic effect of the major allele ('beta GEMMA'), the percentage of variance explained by the SNP ('pve') and its mode as retrieved from the single-SNP analysis.

Table S4. Summary of the GWAS results per trait. All traits reported in this table had at least one significant association with at least one method. The two first columns indicate the number of SNPs found significant with a SNP-by-SNP method (GEMMA) and with a multi-SNP method (MLMM, stepwise regression set to a maximum of 7 steps). The last column indicates the total genotypic variation explained by the set of SNPs retained with MLMM. 'NA', not available.

trait	Nb of significant SNPs GEMMA	pve GEMMA	Nb of significant SNPs MLMM	Nb of significant with both methods	% explained variance MLMM
nb_axes	1	0.128	5	1	0.405
Dim.2	1	0.065	6	1	0.393
c_volume	0	NA	3	0	0.263
TCSA	2	0.151	3	2	0.224
NDVI	13	0.157	2	1	0.217
MCARI2	6	0.216	2	2	0.208
Dim.1	2	0.088	2	1	0.171
ci	1	0.040	2	1	0.132
resp.TSTA_27.07	3	0.188	1	1	0.118
total_length	1	0.101	1	1	0.101
GNDVI	10	0.162	1	1	0.100
TSTA.WW	1	0.098	1	1	0.098
a_volume	2	0.079	1	1	0.079
contrib_17.07	2	0.15	1	1	0.234

Table S5. List of genes underlying the most highly reliable SNPs. *[This large table is provided as a separate Excel file].* We searched for genes in an interval of ±100kb around the highly reliable SNPs. QTL_ID is the unique identifier of the interval. The name of the *Malus domestica* gene (Gene_MD) is indicated together with its chromosome, start and end position in bp. Physical positions are based on the apple genome GDDH13 v1.1. Match (M) indicates the locus name of the orthologue gene in *Arabidopsis thaliana*. The following columns synthetize the information retrieved from TAIR and Uniprot databases: TAIR.Names, names associated with the *Arabidopsis* locus; TAIR.Description and Uniprot.Function; description of the gene function respectively retrieved from TAIR and Uniprot; GO biological process, the GO biological process retrieved from TAIR. In the column Best candidates, we highlighted likely candidates in relation with the trait corresponding to the QTL. The last column corresponds to the raw data extracted from www.rosaceae.org.

Table S6. Co-localizations between associated SNP on the apple core collection and SSR associated on bi-parental populations for similar or related traits. In this comparison, canopy temperature and vegetation indices were compared to similar traits from Virlet *et al.* (2017) and to hydraulic traits from Lauri *et al.* (2011); tree architecture related traits were compared to manually measured architectural traits (from Segura *et al.*, 2007 and 2009). To identify co-localizations, the associated SNPs of the current study were located on the physical map and the flanking GD_ SNPs were located on the integrated map, both on the GDR website (ww.rosaceae.org). Similarly, the SSR markers associated to traits reported in previous studies on bi-parental populations were located on the integrated map and on the genetic map of the bi-parental populations to compare the respective positions of associated SNP- and SSR- based QTLs. Only zones that clearly overlap are listed.

Chr	Traits in GWAS	Associated AX. SNPs	Position on GDDH genome	Flanking GD SNPs	Position (in cM) on integrated map	Associated trait in ''Red Delicious' x 'Granny Smith' population	QTL interval (SSR markers)	Positions (in cM) on Stk x GS map	Positions (in cM) on integrated map	Reference
2	resp.TSTA_ 27.07	AX-115353652	30,732,527	GD_SNP00022	66.7	Slope of 50% hydraulic	CHO5e03 - CH03d01_G	[54-72]	[68.3 - 79.8]	Lauri et al., 2011
				GD_SNP02223	68.6					
4	total_length	AX-105214995	21,791,671	GD_SNP01095	30.00	Nb of branches per tree	CH01d03y - Hi04c10x	[16 - 25]	[19.2 - 31.7]	Segura et al., 2009
				GD_SNP00928	31.00					
13	a_volume, Dim1	AX.115238189	3,297,680	GD_SNP00195	12.6					
				GD_SNP01742	13.1					
13	GNDVI	AX-115431272	4,366,451	GD_SNP00565	19.1	Nb of long branches per tree	Hi04g05 - CH02g01	[0 - 13]	[16.9 - 22.5]	Segura et al., 2007
				GD_02336	[9 - 20] depending on parental map	Dh : Hydraulic vessel diameter	Hi04g05 - Hi04f09	[15.5 - 30.5]	[17.2 - 34.7]	Lauri et al., 2011
				GD_SNP00128	30.9					

References listed in the table:

Lauri P-É, Gorza O, Cochard H, Martinez S, Celton J-M, Ripetti V, Lartaud M, Bry X, Trottier C, Costes E. 2011. Genetic determinism of anatomical and hydraulic traits within an apple progeny. Plant, Cell & Environment 34: 1276–1290.

Segura V, Denancé C, Durel C-E, Costes E. 2007. Wide range QTL analysis for complex architectural traits in a 1-year-old apple progeny. Genome 50: 159–171.

Segura V, Durel C-E, Costes E. 2009. Dissecting apple tree architecture into genetic, ontogenetic and environmental effects: QTL mapping. Tree Genetics & Genomes 5: 165–179.

Table S7. List of allelic effects for the 16 highly reliable associations and the 241 cultivars of the apple tree core-collection. *[This large table is provided as a separate Excel file]*. The three first columns correspond to the identifiers of the 241 cultivars following the assignment of MUNQ (Malus UNiQue genotype) codes to a large set of apple accessions genotyped with common SSR (microsatellite) markers as described in https://doi.org/10.15454/HKGMAS. It contains the following columns: - Accession_Number: Unique identifier of the accession in the collection - Accession_Name: Name of the accession - MUNQ: Malus UNiQue genotype code assigned according to duplicate group (or singleton). The following columns report for each highly reliable SNP (listed in Table 2), the allele carried (0, 1, 2) by each cultivar together with its effect ('effect+' for a positive effect on the trait, 'effect-' for a negative effect on the trait, 'effect+/-' if the cultivar is heterozygous). The 16 SNPs are ordered per type of trait for which they are highly reliable (vegetative architecture, vegetation indices, light interception, canopy surface temperature).
